# Morphine pharmacokinetics and opioid transporter expression at the blood-retina barrier of male and female mice

**DOI:** 10.3389/fphar.2023.1206104

**Published:** 2023-06-14

**Authors:** Casey-Tyler Berezin, Nikolas Bergum, Glenda M. Torres Lopez, Jozsef Vigh

**Affiliations:** ^1^ Cell and Molecular Biology Graduate Program, Colorado State University, Fort Collins, CO, United States; ^2^ Department of Biomedical Sciences, Colorado State University, Fort Collins, CO, United States

**Keywords:** blood-retina barrier, morphine, P-glycoprotein, ABC transporters, sex differences, blood-brain barrier

## Abstract

Opioids are effective analgesics for treating moderate to severe pain, however, their use must be weighed against their dangerous side effects. Investigations into opioid pharmacokinetics provide crucial information regarding both on- and off-target drug effects. Our recent work showed that morphine deposits and accumulates in the mouse retina at higher concentrations than in the brain upon chronic systemic exposure. We also found reduced retinal expression of P-glycoprotein (P-gp), a major opioid extruder at the blood-brain barrier (BBB). Here, we systematically interrogated the expression of three putative opioid transporters at the blood-retina barrier (BRB): P-gp, breast cancer resistance protein (Bcrp) and multidrug resistance protein 2 (Mrp2). Using immunohistochemistry, we found robust expression of P-gp and Bcrp, but not Mrp2, at the inner BRB of the mouse retina. Previous studies have suggested that P-gp expression may be regulated by sex hormones. However, upon acute morphine treatment we found no sex differences in morphine deposition levels in the retina or brain, nor on transporter expression in the retinas of males and females with a high or low estrogen:progesterone ratio. Importantly, we found that P-gp, but not Bcrp, expression significantly correlated with morphine concentration in the retina, suggesting P-gp is the predominant opioid transporter at the BRB. In addition, fluorescence extravasation studies revealed that chronic morphine treatment did not alter the permeability of either the BBB or BRB. Together, these data suggest that reduced P-gp expression mediates retinal morphine accumulation upon systemic delivery, and in turn, potential effects on circadian photoentrainment.

## 1 Introduction

Opioid drugs have been used as effective analgesics for acute and chronic pain for thousands of years (Brownstein, 1993; Benyamin et al., 2008; Williams et al., 2013). However, their use can trigger adverse effects including addiction, sleep/wake disturbances and overdose-related death (Benyamin et al., 2008; Tripathi et al., 2020; Huhn and Finan, 2021). To safely use opioid drugs for pain management, it is crucial to elucidate the pharmacokinetics of opioid drugs in their target tissues (Nafziger and Barkin, 2018).

Opioids exert both their desired (i.e., analgesic) and undesired on-target effects through μ-opioid receptors expressed in the central nervous system (CNS) (Benyamin et al., 2008; Pasternak and Pan, 2013; Schaefer et al., 2017; Bergum et al., 2022c). The movement of opioids from the systemic circulation into their CNS targets is restricted by the blood-brain barrier (BBB) and blood-retina barrier (BRB) (Yang et al., 2018; Liu and Liu, 2019). The integrity of these barriers and the transporters contributing to the movement of opioids across them are therefore critical determinants of opioid pharmacokinetics. ATP-binding cassette (ABC) transporters, expressed by the specialized endothelial cells that constitute blood-CNS barriers, are involved in the efflux of a wide range of metabolites, peptides, and drugs, and are key mediators of opioid drug transport (Yang et al., 2018; Liu and Liu, 2019). Importantly, the efficacy of opioid extrusion by different ABC transporters depends on the specific opioid substrates (Miller, 2015; Yang et al., 2018). Permeability glycoprotein (P-gp) is the best-studied ABC transporter with respect to the prototypical opioid, morphine, and has been considered the most critical for opioid transport in the brain (Xie et al., 1999; Dagenais et al., 2001; Inturrisi, 2002; Loscher and Potschka, 2005; Seleman et al., 2014; Chaves et al., 2017; Schaefer et al., 2017). Nonetheless, others such as breast cancer resistance protein (Bcrp) and multidrug resistance proteins (e.g., Mrp2, Mrp4) may also contribute (Gharavi et al., 2015; Chapy et al., 2016; Yang et al., 2018). Indeed, morphine has been shown to be a substrate for P-gp and Mrps at the BBB, and may also alter the transport activity of P-gp, Mrps, and Bcrp at this barrier (Yang et al., 2018). Furthermore, P-gp and Mrps appear to be involved in the transport of the endogenous opioid peptide, β-endorphin (King et al., 2001; Su, 2002; Su and Pasternak, 2013; Yang et al., 2018).

We recently observed that morphine deposits and accumulates in the mouse retina at higher concentrations than the brain following systemic administration (Bergum et al., 2022a). Using quantitative reverse transcription PCR (qRT-PCR) we also found that P-gp mRNA expression is significantly lower in the retina than the brain (Bergum et al., 2022a). These findings are consistent with reports of reduced P-gp function at the BRB compared to the BBB in both rodents (Fujii et al., 2014; Chapy et al., 2016; Liu and Liu, 2019) and humans (Bauer et al., 2017). Despite evidence that P-gp transports opioids across the BBB and is functional at the BRB, the contributions of ABC transporters at the BRB to opioid pharmacokinetics in the retina are relatively unknown (Bergum et al., 2022a).

Furthermore, basic and preclinical research studies have only recently begun to examine the role of sex (i.e., biological sex, estrus/menstrual cycle, and sex hormones) on drug pharmacokinetics and pharmacodynamics. These studies are essential as women experience chronic pain with greater frequency and duration compared to men (Bartley and Fillingim, 2013). In rodents, males are more responsive to opioid-induced analgesia compared to female mice (Candido et al., 1992; Gabel et al., 2021). While this effect has been primarily attributed to sex-dependent differences in opioid receptor expression (Loyd et al., 2008; Guajardo et al., 2017), the role of sex/sex hormones on opioid metabolism and pharmacokinetics warrants further research (Gabel et al., 2021). Several studies have suggested that ABC transporter (i.e., P-gp and Bcrp) expression is higher in females than males, particularly in the liver (Piquette-Miller et al., 1998; Tanaka et al., 2005; Suzuki et al., 2006; Shchul’kin et al., 2017). However, efforts to determine how sex differences in transporter expression affect the behavioral outcomes of opioids are complicated by tissue-, substrate- and species-dependent expression and function (Tanaka et al., 2005; Bebawy and Chetty, 2009; Robison et al., 2019; Dalla et al., 2022).

Here, we aimed to characterize which putative opioid transporters (P-gp, Bcrp, and Mrp2) expressed at the inner BRB (iBRB) of male and female mice, and to elucidate their contributions to morphine pharmacokinetics in the mouse retina. We quantified transporter expression and morphine deposition in the retina and brain by qRT-PCR and liquid chromatography-tandem mass spectrometry (LC-MS/MS), respectively, 1 h after systemic morphine administration. Although the expression of P-gp and Bcrp was higher in female brains than male brains, we found no differences in transporter expression or morphine deposition in the retina between male mice, females with a high estrogen/progesterone ratio (High E/P; proestrus and estrus) and females with a low estrogen/progesterone ratio (Low E/P; metestrus and diestrus). Our results suggest that P-gp and Bcrp, but not Mrp2, are positioned to play a significant role in the transport of morphine at the BRB. In addition, we show that P-gp, but not Bcrp, expression significantly correlates with morphine content in the retina. Furthermore, we found that chronic morphine exposure does not affect BBB or BRB permeability. Thus, the findings presented here highlight P-gp as a significant regulator of morphine pharmacokinetics, not only at the BBB, but at the BRB of both male and female mice.

## 2 Results

### 2.1 P-gp and Bcrp, but not Mrp2, may transport morphine at the BRB

**FIGURE 1:**
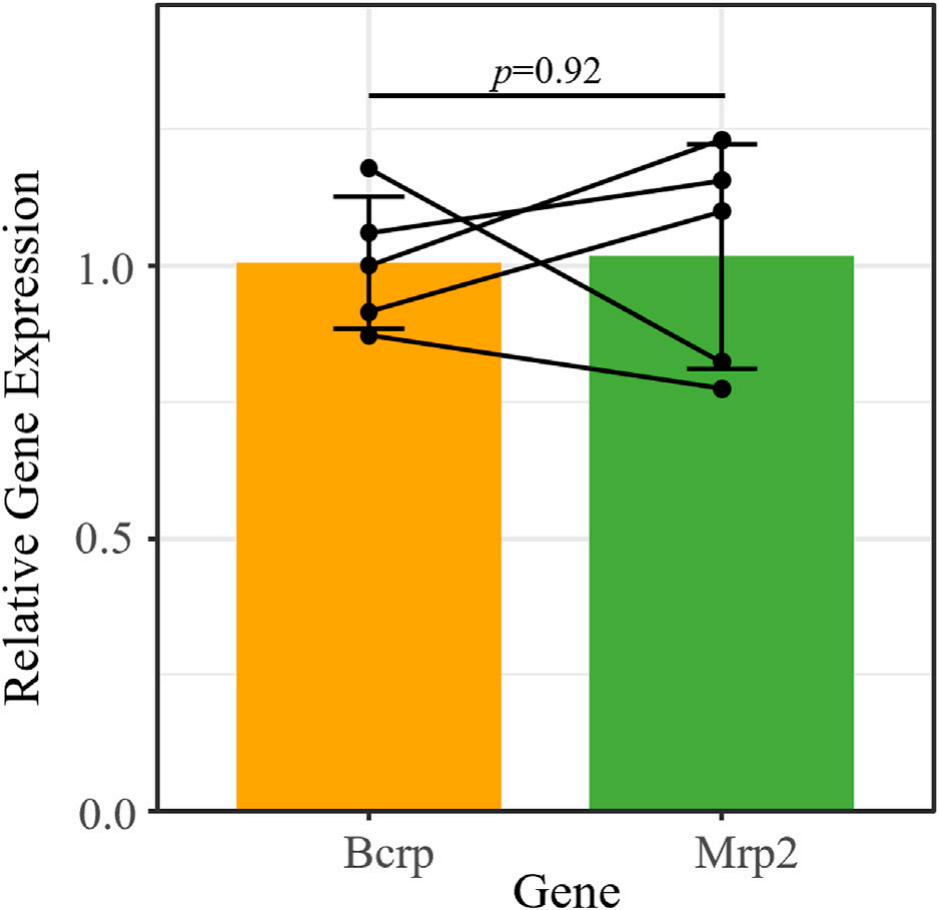
Bcrp and Mrp2 have similar mRNA expression levels in the mouse retina. *Bcrp* and *Mrp2* mRNA expression quantified by qRT-PCR, relative to the reference genes *Tbp* and *β-actin*. Lines connect samples from the same animal. Data presented as mean ± SD. Paired *t*-test, t (4) = −0.11, *p* = 0.92.

We characterized the expression of three ABC transporters in the retina: P-gp (*Mdr1a*/*Abcb1a*), Bcrp (*Abcg2*), and Mrp2 (*Abcc2*). We previously reported that P-gp mRNA is expressed in the retinas of mice that received chronic morphine (20 mg/kg for 6 d, twice daily) or vehicle (saline) via intraperitoneal (i.p.) injections (Bergum et al., 2022a). Using the RNA extracted from those same saline-treated retinas (*n* = 5), we performed qRT-PCR to measure Bcrp and Mrp2 expression relative to the reference genes *β-actin* and *Tbp* (Figure 1). We confirmed that Bcrp (relative gene expression [RGE] mean ± s.d.: 1.01 ± 0.12) and Mrp2 (1.02 ± 0.2) mRNA are detectable in the whole mouse retina at similar levels (t (4) = −0.11, *p* = 0.92; Figure 1).

**FIGURE 2:**
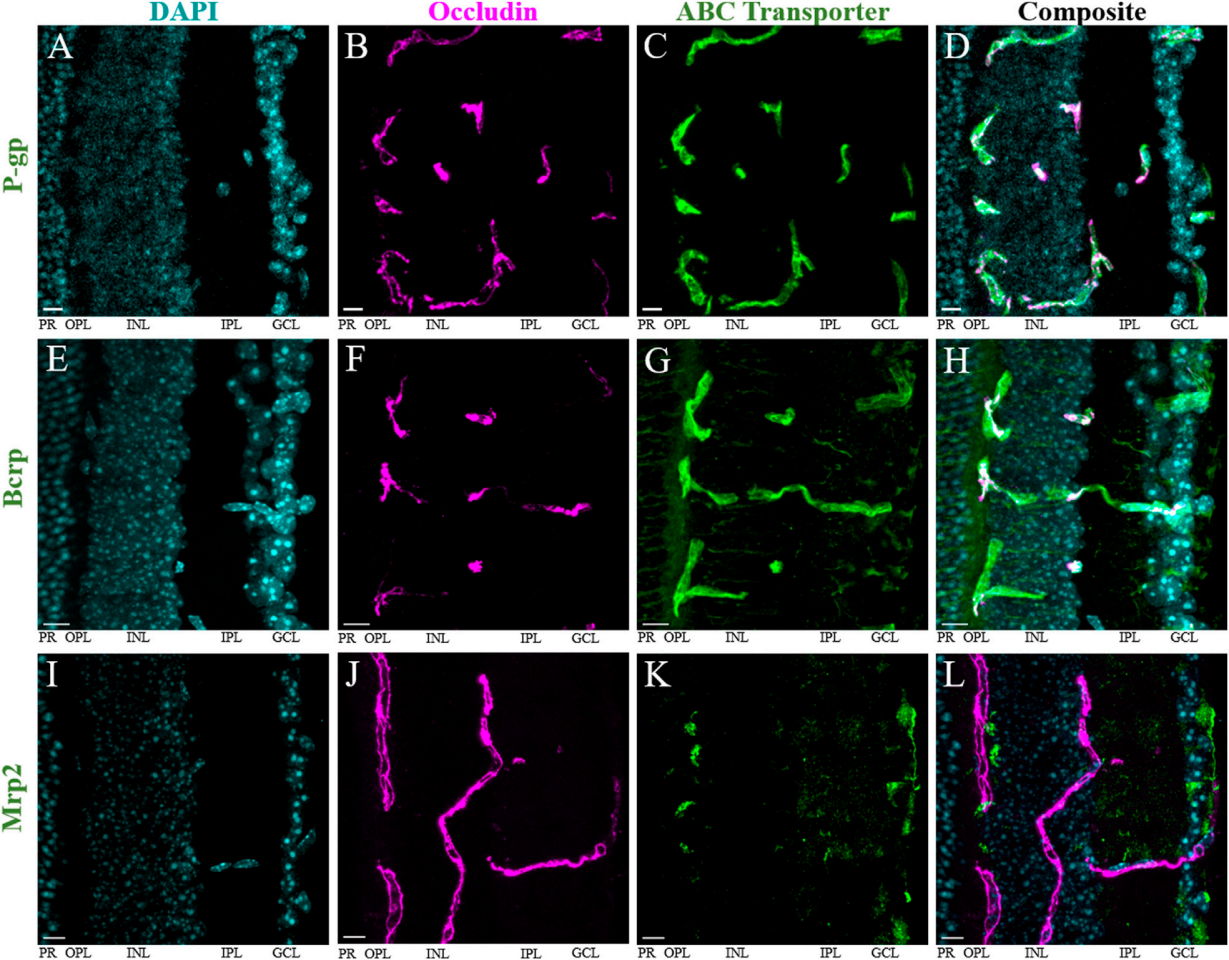
P-gp and Bcrp, but not Mrp2, are primarily expressed at the iBRB. Immunohistochemistry in retinal cryosections reveals robust expression of **(A–D)** P-gp and **(E–H)** Bcrp, but not Mrp2 **(I–L)**, at the inner blood-retina barrier (iBRB). DAPI (blue) labels nuclei within the retina **(A,E,I)**, occludin (magenta, pseudocolored) labels tight junctions between endothelial cells that make up the iBRB **(B,F,J)**. All images are maximum projections of all Z-stack images containing the tissue. The average Pearson’s correlation coefficient (*r*) of colocalization of the transporter with occludin was **(A–D)** 0.42 ± 0.16 for P-gp; **(E–H)** 0.46 ± 0.1 for Bcrp; and **(I–L)** 0.10 ± 0.07 in regions of positive colocalization of Mrp2 and occludin, and −0.006 ± 0.03 in regions of negative colocalization of Mrp2 and occludin. Scale bars = 10 μm. GCL = ganglion cell layer, IPL = inner plexiform layer, INL = inner nuclear layer, OPL = outer plexiform layer, PR = photoreceptor layer.

We then performed immunohistochemistry (IHC) with fluorescence confocal microscopy to evaluate P-gp, Bcrp and Mrp2 expression at the iBRB of male and female mice (Figure 2). The iBRB was labeled using an antibody against occludin, a marker for the tight junctions of the endothelial cells which make up the iBRB (Morcos et al., 2001). The iBRB extends from the inner capillary bed in the ganglion cell layer (GCL), through the inner plexiform layer (IPL) and inner nuclear layer (INL), to the outer capillary bed in the outer plexiform layer (OPL) (Figure 2). The literature suggests that both endogenous opioid peptides and their receptors are predominantly expressed in the inner retina (Wamsley et al., 1981; Altschuler et al., 1982; Gallagher et al., 2010; Cleymaet et al., 2019; Berezin et al., 2022). Previous work has also suggested that P-gp and Bcrp are not expressed at the outer BRB (Chapy et al., 2016). Therefore, the outer BRB, which is established by the retinal pigment epithelium, was not analyzed in this study.

Immunolabeling of occludin and P-gp was performed in both retinal cryosections (*n* = 4, Figures 2A–D) and whole-mount preparations (*n* = 6, Supplementary Figures S1A–C) from male and female mice. The anti-occludin antibody produced robust labeling of vascular endothelium in both preparations. In cryosections, we found extensive P-gp immunolabeling exclusively co-localizing with that of occludin in the iBRB (Figures 2A–D). However, in the whole-mount retinas, we commonly observed regions of the occludin-positive vasculature with strong and some with weaker P-gp co-labeling (Supplementary Figures S1A–C). Across all the images obtained from both preparations (4–6/mouse, *n* = 51), the average Pearson’s correlation coefficient (*r*) was 0.32 ± 0.16 (mean ± s.d.). Using Costes’ automatic threshold and randomization procedures, we found this to represent true colocalization in all cases (*p* = 100%) (Costes et al., 2004). Colocalization parameters (*r*, Mander’s coefficients M_1_ and M_2_) were then averaged across all images obtained from a single mouse. Consistent with the observed P-gp immunolabeling patterns, the average *r* was significantly lower in whole-mount retinas (0.24 ± 0.12) than cryosections (0.42 ± 0.16) (*p* = 0.04, *t*-test, t (6.4) = 2.57; Supplementary Figure S1D). There was no significant difference in the Mander’s colocalization coefficient M_1_ (indicating the extent of occludin-labeled area overlapping with P-gp) (Manders et al., 1993) between whole-mounts (0.20 ± 0.29) compared to the cryosections (0.41 ± 0.31) (*p* = 0.1, *t*-test, t (7.4) = 1.9; Supplementary Figure S1E). Similarly, there was no significant difference in the M_2_ coefficient (indicating the extent to which P-gp overlaps with occludin) between whole-mount (0.16 ± 0.21) and cryosection preparations (0.30 ± 0.31) (*p* = 0.196, *t*-test, t (5.6) = 1.5; Supplementary Figure S1F). These results are consistent with the qualitative differences in P-gp immunostaining described above, and support the idea that while the level of P-gp expression may vary throughout the vasculature, P-gp expression in the mouse retina is confined to blood vessels. Importantly, we found no significant qualitative or quantitative (*r*, M_1_, or M_2_) differences between male and female mice (*n* = 7 and 3, respectively) (*p* > 0.05, *t*-test; Supplementary Figures S2A–C).

Bcrp immunolabeling in retinal cryosections from male and female mice (*n* = 7) also revealed robust expression of Bcrp in occludin-labeled iBRB blood vessels (Figures 2E–H). However, Bcrp labelling was not restricted to iBRB, but found in other structures in the inner retina and photoreceptor layer as well. Outside blood vessels, the anti-Bcrp antibody appeared to label neuronal projections, as well as a diffuse pattern in the OPL and GCL (Figures 2E–H). Across all images (6/mouse, *n* = 42), the average *r* was 0.46 ± 0.1. We found true colocalization in all images (*p* = 100%) (Costes et al., 2004). Colocalization parameters (*r*, Mander’s coefficients M_1_ and M_2_) were then averaged across all images obtained from a single mouse. Consistent with diffuse Bcrp staining in the inner retina, the average M_1_ coefficient (indicating the extent of occludin-labeled area overlapping with Bcrp) was high (0.74 ± 0.13) compared to our results for P-gp. Conversely, the average M_2_ coefficient (indicating the extent to which Bcrp overlaps with occludin) was more similar to the results for P-gp at 0.27 ± 0.09. As with P-gp, we found no significant qualitative or quantitative (*r*, M_1_, or M_2_) differences between male and female mice (*n* = 4 and 3, respectively) (*p* > 0.05, *t*-test; Supplementary Figures S2D–F). Despite the presence of Bcrp in some retinal cells outside the iBRB, its robust expression throughout the vasculature suggests it could be a significant transporter at the mouse iBRB.

**FIGURE 3:**
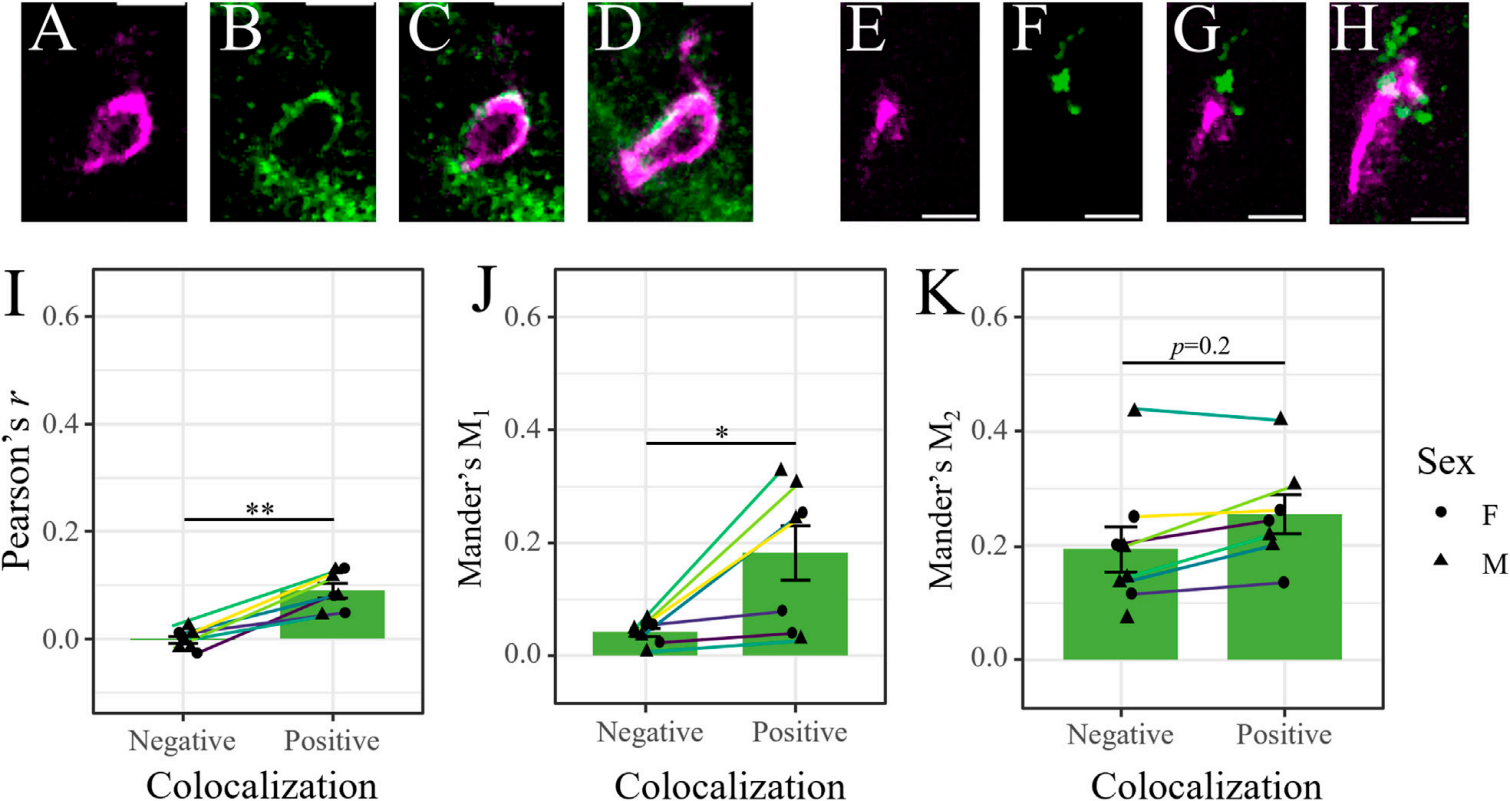
Mrp2 expression in the retina is not exclusive to the iBRB. **(A–D)** Representative image of positive colocalization (*p*>95%, Costes’ methods) of occludin (magenta [pseudocolored]), **(A)** and Mrp2 (green, **(B)** in a single optical section. Merged signals in **(C)** a single optical section and **(D)** in a maximum projection image through the thickness of the section. Image reveals Mrp2 expression overlapping an occludin-labeled retinal blood vessel. **(E–H)** Representative image of negative colocalization of occludin and Mrp2 (*p*<95%, Costes’ methods), presented as in **(A–D)**. Image reveals that Mrp2 immunolabeling is often near, but not overlapping, occludin-labeled retinal blood vessels. Scale bars = 5 μm. **(I–K)** Colocalization parameters for instances of positive and negative colocalization in male (triangles) and female (circles) mice. Each point represents the average value across all positively/negatively colocalizing regions obtained from a single mouse; lines connect values from the same mouse (*n* = 55 positive and 90 negative regions across 8 mice, 13–22 regions/mouse). **(I)** Pearson’s correlation coefficient (*r*) and **(J)** Mander’s M_1_ coefficient (indicating the extent of occludin-labeled area overlapping with Mrp2) were significantly higher in positively colocalizing regions. We found no difference in **(K)** Mander’s M_2_ coefficient (indicating the extent of Mrp2-labeled area overlapping with occludin). Data presented as mean ± SEM. Paired t-tests *p < 0.05 **p < 0.01.

Mrp2 immunostaining in retinal cryosections (*n* = 8) from male and female mice did not result in robust colocalization with occludin-labeled blood vessels (Figures 2I–L). Rather, it primarily labeled structures at the proximal INL, OPL and GCL, in addition to diffuse labeling of the IPL (Figures 2I–L). There were some imaged regions where Mrp2 appeared to overlap with occludin-labeled blood vessels, which we isolated for colocalization analysis (13–22/mouse, *n* = 145) (Figures 3A–H). We found true colocalization (*p* > 95%) in about a third of the 145 regions analyzed (Costes et al., 2004). Colocalization parameters (*r*, Mander’s coefficients M_1_ and M_2_) were then averaged across all negatively or positively colocalizing regions obtained from a single mouse. After accounting for paired samples (i.e., one mouse has both positive and negative regions), the average *r* in regions with negative colocalization (*n* = 90, −0.006 ± 0.03, Figures 3E–H) was significantly lower than in images with positive colocalization (*n* = 55, 0.10 ± 0.07, Figures 3A–D) (*p* = 0.0012, paired *t*-test, t (7) = -5.25; Figure 3I). Accordingly, the M_1_ coefficient (indicating the amount of occludin overlapping Mrp2) was significantly lower in regions with no colocalization (0.04 ± 0.07) than in positive regions (0.23 ± 0.16) (*p* = 0.04, paired *t*-test, t (7) = −2.52; Figure 3J). The M_2_ coefficient (indicating the amount of Mrp2 overlapping occludin) was not significantly different between images without colocalization (0.187 ± 0.166) than with colocalization (0.24 ± 0.14) (*p* = 0.18, paired *t*-test, t (7) = −1.5; Figure 3K), likely due to diffuse Mrp2 labeling. As with P-gp and Bcrp, we found no significant qualitative or quantitative (*r*, M_1_, or M_2_) differences in positively colocalizing regions between male and female mice (*n* = 5 and 3, respectively) (*p* > 0.05, *t*-test; Supplementary Figures S2G–I). Despite Mrp2’s occasional presence at the iBRB, its expression primarily by other cell types suggests that its principal function in the retina is not the extrusion of substrates from vascular endothelial cells. Thus, we chose to focus only on P-gp and Bcrp in our subsequent analyses.

### 2.2 Morphine deposits similarly in the brain and retina of male and female mice

**FIGURE 4:**
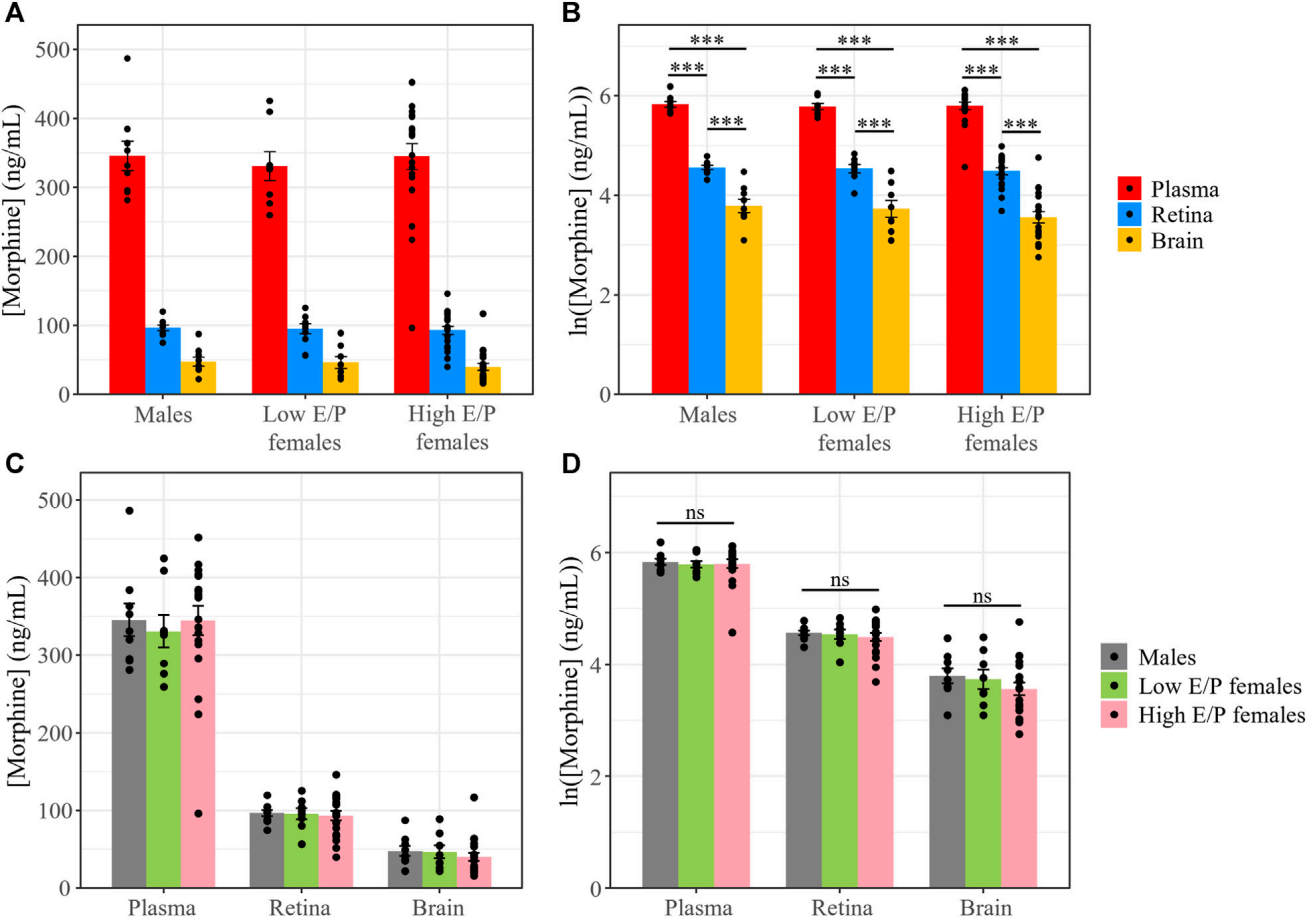
Morphine concentrations in the plasma, retina and brain are unaffected by differences in sex/sex hormones. **(A)** Plasma, retina, and brain morphine concentrations and **(B)** natural logarithmic (ln) transformed morphine concentrations collected from mice sacrificed 1 h following a 20 mg/kg i.p. morphine injection at light onset (ZT 0). **(C,D)** No significant differences exist in morphine deposition in any tissue between male and female mice. Data presented as mean ± SEM. Two-way ANOVA with Tukey *post hoc* adjustments was performed on the ln scale. ns = not significant (*p* > 0.05)*,* *** = *p <* 0.0001.

We previously investigated the pharmacokinetics of morphine in the serum, brain and retina of male mice over 11 h after systemic morphine administration (20 mg/kg, i.p.) (Bergum et al., 2022a). Morphine content peaked in the serum, brain and retina 1 h following injection (Bergum et al., 2022a). Here, we investigated whether morphine deposition at this time point differs between the sexes and/or between females in different estrous stages. Male mice (*n* = 9), as well as females with a low estrogen:progesterone ratio (Low E/P; *n* = 8) and High E/P females (*n* = 20) received a single injection of morphine (20 mg/kg, i.p.) and were sacrificed 1 h later. The mean weight in the male group was 26.9 ± 1.82 g, 21.4 ± 1.17 g in the High E/P female group, and 20.8 ± 0.81 g in the Low E/P female group. Raw morphine content (ng/mL) in the plasma, brain, and retina was quantified by LC-MS/MS. The raw concentrations detected in the male mice in this study were comparable to our previously published data (Bergum et al., 2022a), with an average concentration in the brain of 47.7 ± 19.3 ng/mL (mean ± SD), 96.6 ± 12.7 ng/mL in the retina, and 346 ± 63.2 ng/mL in the plasma detected here. The data were log_e_ (ln) transformed to satisfy the assumptions of ANOVA. We found that tissue type, but not sex/estrus group (*F* = 0.68, *p* = 0.51), was a significant predictor of morphine content (*F* = 378, *p* < 2e-16). Raw morphine content was significantly higher in the plasma than retina in all groups: males (t (68) = 8.8, *p* < 0.0001), Low E/P females (t (68) = 8.2, *p* < 0.0001) and High E/P females (t (68) = 13.5. *p* < 0.0001) (Figure 4). Furthermore, raw morphine content was significantly higher in the retina than in the brain in all groups: males (t (68) = 5.3, *p* < 0.0001), Low E/P females (t (68) = 5.2, *p* < 0.0001) and High E/P females (t (68) = 9.6, *p* < 0.0001) (Figure 4). We found no significant sex or estrous stage differences (*p* > 0.05) in morphine deposition in the plasma, retina, or brain (Figure 4).

**FIGURE 5:**
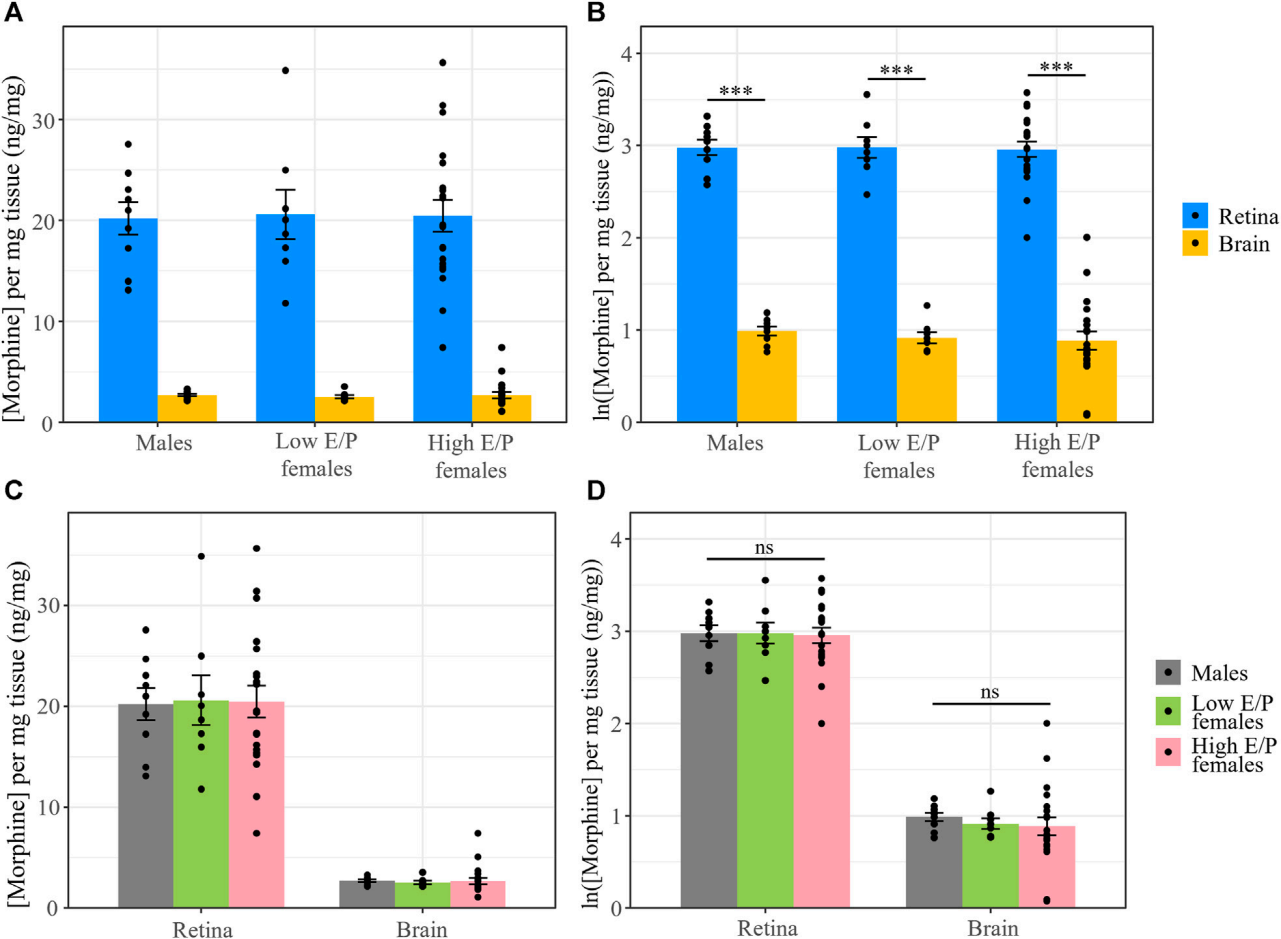
Normalized morphine concentrations in the retina and brain are the same across male and female mice. **(A)** Morphine concentrations normalized to the weight of the tissue and **(B)** natural logarithmic (ln) transformed normalized morphine concentrations collected from mice sacrificed 1 h following a 20 mg/kg i.p. morphine injection at light onset (ZT 0) show retinal morphine levels exceed brain levels. **(C,D)** No significant differences exist in normalized morphine concentrations in the retina and brain between male and female mice. Data presented as mean ± SEM. Data were ln transformed to satisfy the assumptions for two-way ANOVA with Tukey *post hoc* adjustments. ns = not significant (*p* > 0.05)*,* *** = *p <* 0.0001.

To account for differences in the tissue weight of each harvested brain/retina sample, we normalized morphine content in the retina and brain to the tissue weight (average of 4.76 ± 0.74 mg and 17.4 ± 8.77 mg, respectively). The data were again ln transformed for statistical analysis. As before, only tissue type was a significant factor (*F* = 1421, *p* < 2e-16, ANOVA). Morphine content in the retina was again significantly higher than in the brain in male mice (t (34) = 19.657, *p* < 0.0001), Low E/P females (t (34) = 19.218, *p* < 0.0001), and High E/P females (t (34) = 30.495, *p* < 0.0001) (Figure 5). No significant sex differences were found in morphine content in the retina or the brain (*p* > 0.05) (Figure 5).

### 2.3 Chronic morphine treatment does not affect opioid transporter expression at the iBRB

We previously reported that P-gp expression in the retina and brain was not significantly different between male mice that received chronic morphine or vehicle (saline) (Bergum et al., 2022a), consistent with reports that elevated P-gp expression after morphine exposure can be attributed to a withdrawal response rather than the morphine treatment itself (Yousif et al., 2012; Chaves et al., 2016). Here, we tested whether Bcrp expression is influenced by chronic morphine exposure (20 mg/kg for 6 days, twice daily) (*n* = 7) compared to vehicle (saline, *n* = 9). All animals were sacrificed 1 h following the last injection. Expression of Bcrp, relative to the reference genes *β-actin* and *Tbp*, was calculated using the saline-treated retinas as the control group. The data were log_2_ transformed to meet the assumptions of ANOVA. Tissue type, but not treatment, was a significant predictor of Bcrp expression (*F* = 96, *p* = 1.18e-7).

**FIGURE 6:**
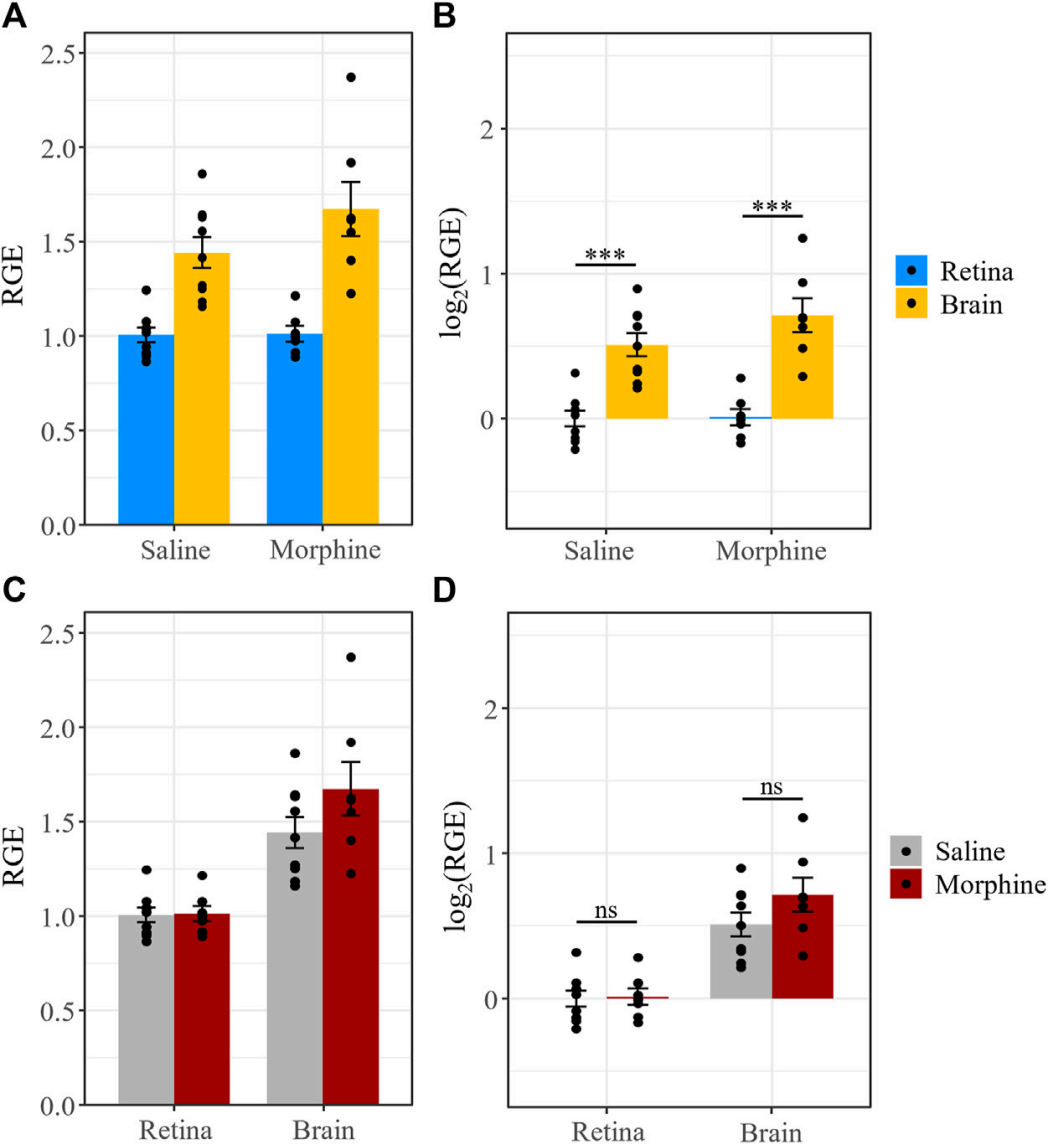
Chronic morphine treatment does not affect Bcrp expression in the retina or brain. **(A)** Bcrp relative gene expression (RGE) and **(B)** log_2_ transformed RGE values are lower in the retina than in the brain 1 h after the last injection of a chronic morphine paradigm (20 mg/kg i.p., twice daily for 6 d). Bcrp mRNA was quantified by qRT-PCR, relative to the reference genes *Tbp* and *β-actin*. **(C,D)** Chronic treatment with morphine does not alter Bcrp expression in the retina or brain relative to vehicle (saline) treatment. Data are presented as the mean ± SEM. Data were log_2_ transformed to satisfy the assumptions for two-way ANOVA with Tukey *post hoc* adjustments. ns = not significant (*p* > 0.05)*,* *** = *p <* 0.0001.

Similar to P-gp (Bergum et al., 2022a), Bcrp expression was lower in the retina than the brain in both the saline-treated (t (14) = −6.24, *p* < 0.0001) and morphine-treated groups (t (14) = −7.58, *p* < 0.0001) (Figure 6). Moreover, we found no significant differences between morphine- and saline-treated mice in terms of Bcrp expression in the retina (t (24.3) = −0.1, *p* = 0.92) and brain (t (24.3) = −1.8, *p* = 0.08) (Figure 6). Thus, neither P-gp nor Bcrp expression is expected to be altered by morphine administration.

### 2.4 Opioid transporter expression at the iBRB is not dependent on sex/hormonal differences

We next investigated whether P-gp and Bcrp expression differs between the sexes and/or between female estrous stages in the context of acute morphine treatment (1 h after injection). Expression of P-gp and Bcrp, relative to the reference genes *β-actin* and *Tbp*, in the brains and retinas of acute morphine-treated males (*n* = 8), High E/P females (*n* = 11), and Low E/P females (*n* = 5) was quantified by qRT-PCR using the male retinas as the control group. The relative gene expression (RGE) values were transformed on the log_2_ scale to satisfy the assumptions of ANOVA. We found that Gene (*F* = 253, *p* < 2e-16), Tissue (*F* = 509, *p* < 2e-16) and Group (*F* = 4.3, *p* = 0.027) were all significant predictors of RGE values, as were the interactions of Gene and Tissue (*F* = 170, *p* < 2e-16) and of Tissue and Group (*F* = 4.8, *p* = 0.011).

**FIGURE 7:**
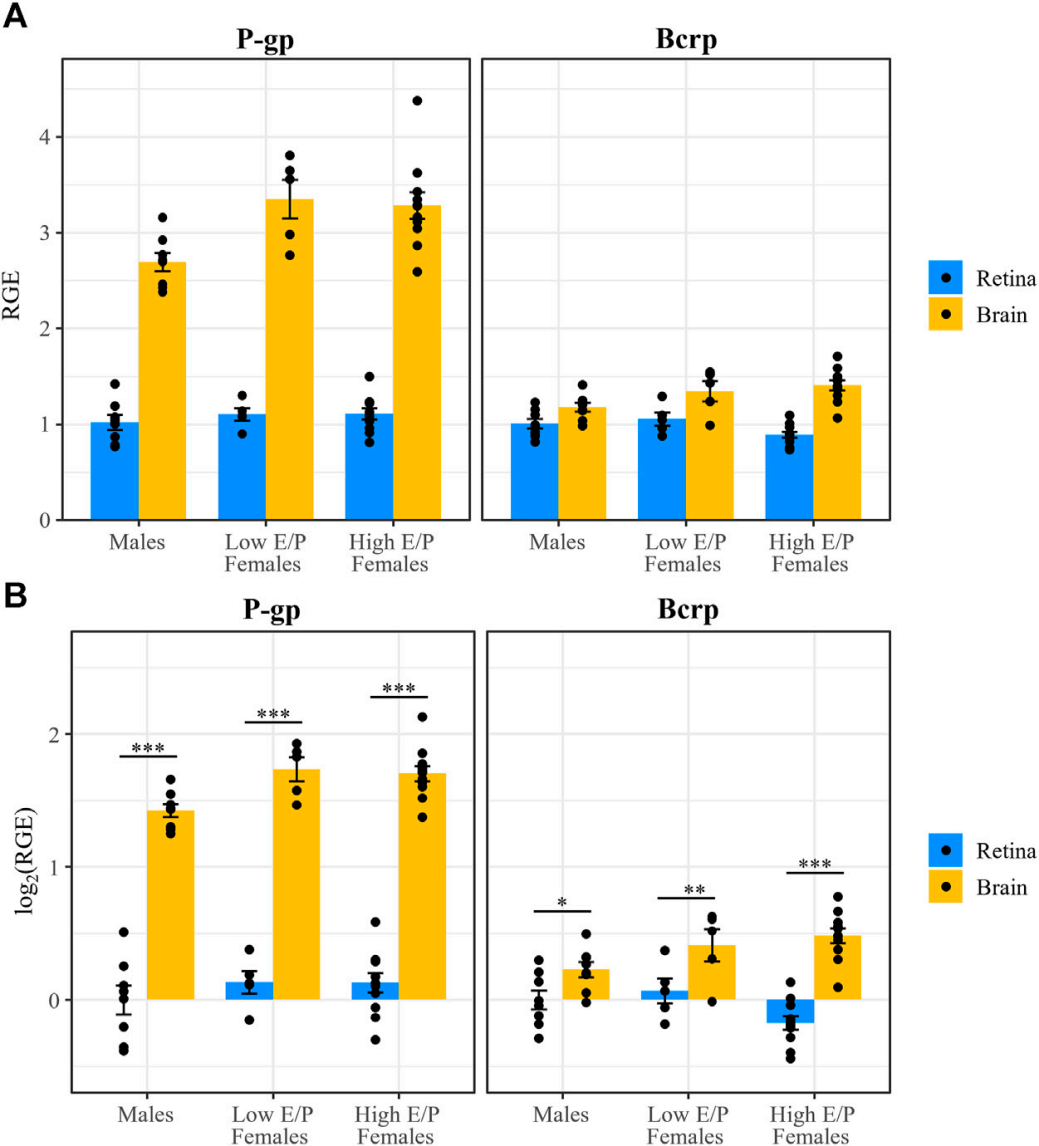
P-gp and Bcrp expression are lower in the retina than brain in male and female mice. **(A)** P-gp and Bcrp relative gene expression (RGE) and **(B)** log_2_ transformed RGE values are lower in the retina than in the brain 1 h following a morphine injection (20 mg/kg i.p.). P-gp and Bcrp mRNA were quantified by qRT-PCR, relative to the reference genes *Tbp* and *β-actin*. Data are presented as the mean ± SEM. Data were log_2_ transformed to satisfy the assumptions for two-way ANOVA with Tukey *post hoc* adjustments. *p < 0.05 **p < 0.01 ***p < 0.0001.

As we showed in chronically treated male mice (Bergum et al., 2022a), P-gp expression was significantly lower in the retina than the brain in all groups: males (t (63) = −14.219, *p* < 0.0001), Low E/P females (t (63) = −12.66, *p* < 0.0001), and High E/P females (t (63) = −18.422, *p* < 0.0001) (Figure 7). Similarly, Bcrp expression was significantly lower in the retina than the brain in all groups: males (t (63) = −2.287), *p* = 0.0255), Low E/P females (t (63) = −2.714, *p* = 0.0086), and High E/P females (t (63) = −7.695, *p* < 0.0001) (Figure 7).

**FIGURE 8:**
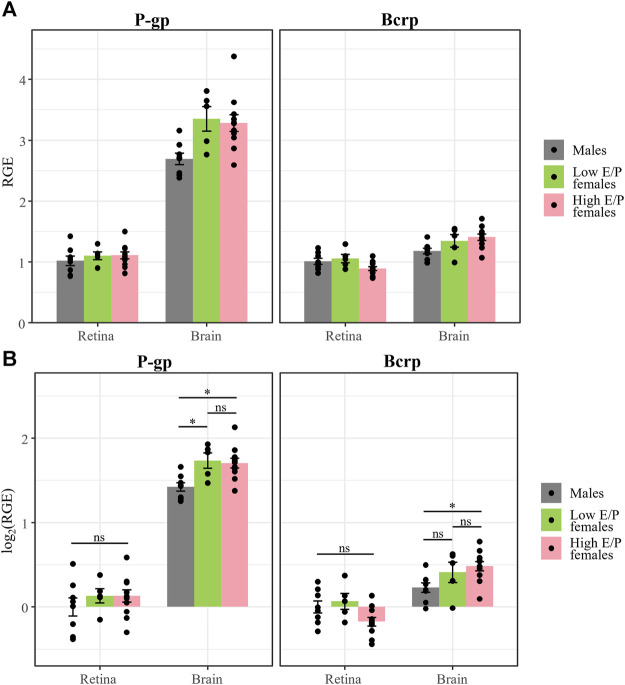
P-gp and Bcrp expression in the retina show no effect of sex or estrous stage. **(A)** P-gp and Bcrp relative gene expression (RGE) and **(B)** log_2_ transformed RGE values show no differences between male and female animals (regardless of estrus stage) 1 h following a morphine injection (20 mg/kg i.p.). Relative Gene Expression (RGE) of Pgp and Bcrp mRNA was quantified by qRT-PCR, relative to the reference genes *Tbp* and *B-actin*. Data are presented as the mean ± SEM. Data were log_2_ transformed to satisfy the assumptions for two-way ANOVA with Tukey *post hoc* adjustments. ns = not significant (*p* > 0.05)*,* *p < 0.05.

In the brain, P-gp expression was significantly lower in males than in both Low E/P females (t (83) = -2.633, *p* = 0.027) and High E/P females (t (83) = -2.909, *p* = 0.0128) (Figure 8). The two female groups were not significantly different from one another (t (83) = 0.28, *p* = 0.9585) (Figure 8). As for Bcrp in the brain, we found that expression in males was significantly lower than High E/P females (t (83) = -2.63, *p* = 0.0272), but not significantly different than Low E/P females (t (83) = −1.5, *p* = 0.2830) (Figure 8). The two female groups were not significantly different from one another (t (83) = −0.65, *p* = 0.7926) (Figure 8). Together, these results are consistent with sex hormones influencing opioid transporter expression in some tissues, such as the brain (Tanaka et al., 2005; Suzuki et al., 2006; Bebawy and Chetty, 2009; Shchul’kin et al., 2017; Dalla et al., 2022).

Conversely, there was no effect of sex or estrous stage on retinal P-gp expression, as expression in males was not significantly different than Low E/P females (t (83) = −1.11, *p* = .51) or High E/P females (t (83) = −1.36, *p* = 0.37), nor were Low E/P and High E/P females significantly different from one another (t (83) = 0.005, *p* = 1.0) (Figure 8). Furthermore, we found no effect of sex or estrous stage on retinal Bcrp expression; expression in males was not significantly different than Low E/P females (t (83) = −0.56, *p* = 0.84) or High E/P females (t (83) = 1.82, *p* = 0.17), nor were Low E/P and High E/P females significantly different from one another (t (83) = 2.155, *p* = 0.085) (Figure 8). Thus, circulating sex hormones do not appear to significantly impact opioid transporter expression at the iBRB.

### 2.5 P-gp is a significant predictor of morphine deposition in the retina

**FIGURE 9:**
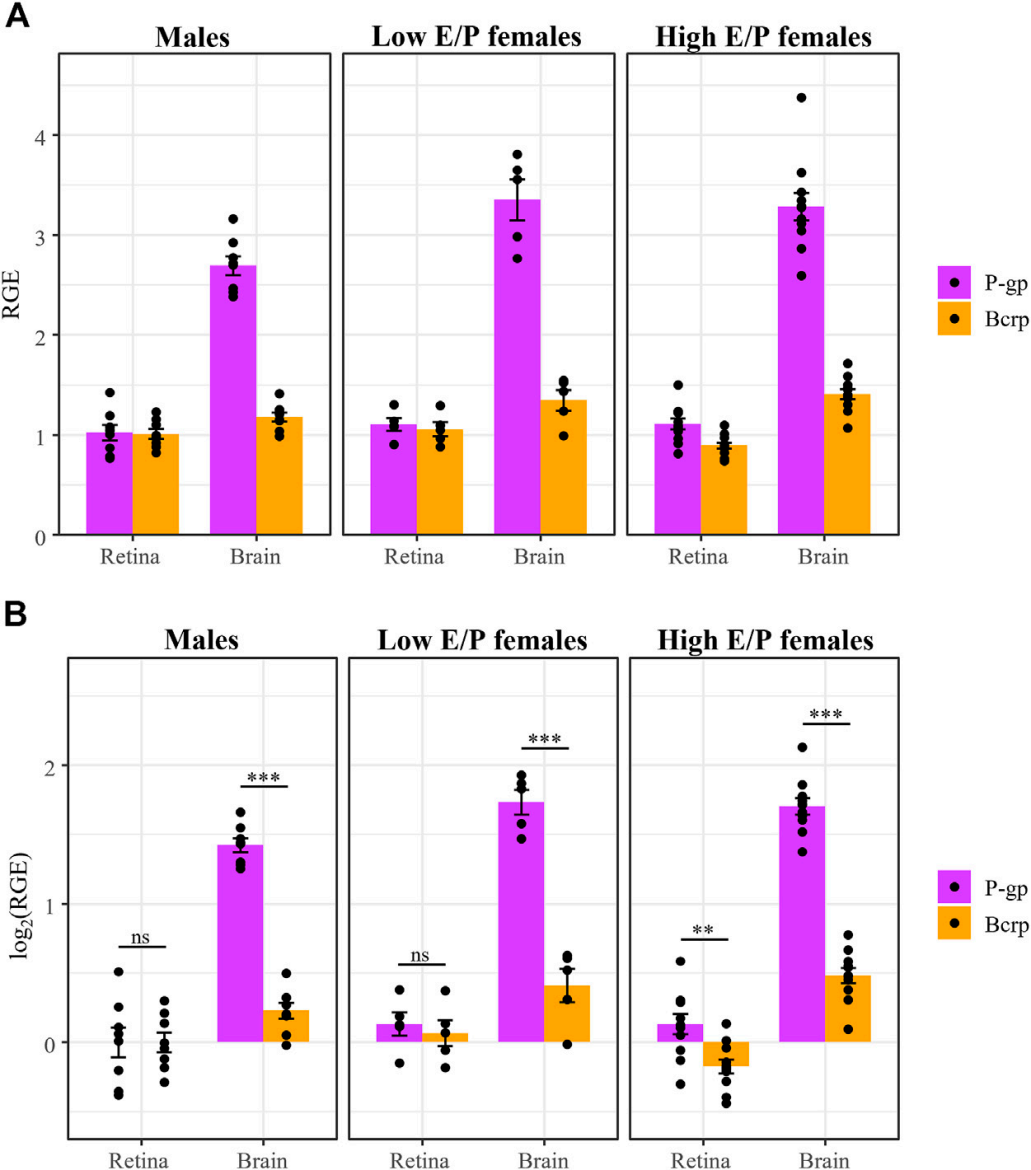
P-gp mRNA expression is higher than Bcrp in the brain, but not in the retina, in both male and female mice. **(A)** P-gp and Bcrp relative gene expression (RGE) and **(B)** log_2_ transformed RGE values show increased P-gp expression compared to Bcrp in the brains of male and female mice 1 h following a morphine injection (20 mg/kg i.p.). Contrastingly, there is no difference in P-gp and Bcrp mRNA expression in the retina of male and Low E/P female mice. P-gp and Bcrp mRNA expression was quantified by qRT-PCR, relative to the reference genes *Tbp* and *β-actin*. Data are presented as the mean ± SEM. Data were log_2_ transformed to satisfy the assumptions for two-way ANOVA with Tukey *post hoc* adjustments. ns = not significant (*p* > 0.05)*,* **p < 0.001, ***p < 0.0001.

Our experimental design allowed for statistical comparison of P-gp and Bcrp expression in the retina and the brain. In the brain, we found a staggering three-fold difference in P-gp expression compared to Bcrp expression in all groups: males (t (63) = 11.9, *p* < 0.0001, ANOVA), Low E/P females (t (63) = 10.46, *p* < 0.0001) and High E/P females (t (63) = 14.306, *p* < 0.0001) (Figure 9). Interestingly, in the retina, the expression of the two transporters was more similar. While P-gp expression was greater than Bcrp expression in High E/P females (t (63) = 3.58, *p* = 0.0007), we found no such significant differences in males (t (63) = 0, *p* = 1.0) or Low E/P females (t (63) = .516, *p* = 0.61) (Figure 9).

**FIGURE 10:**
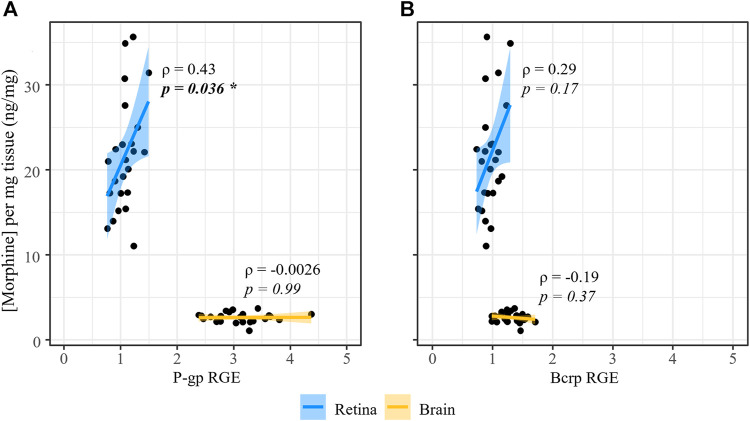
P-gp expression at the iBRB is significantly correlated to morphine deposition in the retina. Spearman’s rank correlation (rho, ρ) was calculated for the relationship between normalized morphine concentration and relative **(A)** P-gp or **(B)** Bcrp mRNA expression in the retina and brain of male and female mice.

We then examined the relationship between transporter expression in the retina or brain and the level of morphine deposition in that tissue, combining our male and female groups (*n* = 24) given the lack of sex/estrous effects on either parameter in the retina. Relative gene expression values and normalized morphine content (ng/mg) were left untransformed, and Spearman’s non-parametric rank correlation rho (ρ) was calculated for each gene in each tissue. In the brain, we found a weak correlation between transporter expression and morphine deposition. The correlation coefficient, ρ, between P-gp and morphine content was −0.0026 (*p* = 0.99), while for Bcrp it was −0.19 (*p* = 0.37). Conversely, we found stronger correlations between transporter expression and morphine content in the retina. For Bcrp, ρ was 0.29 (*p* = 0.17), while for P-gp we found a statistically significant correlation between expression and morphine deposition (*ρ* = 0.43, *p* = 0.036) (Figure 10).

### 2.6 Chronic morphine treatment does not affect BBB or BRB permeability

**FIGURE 11:**
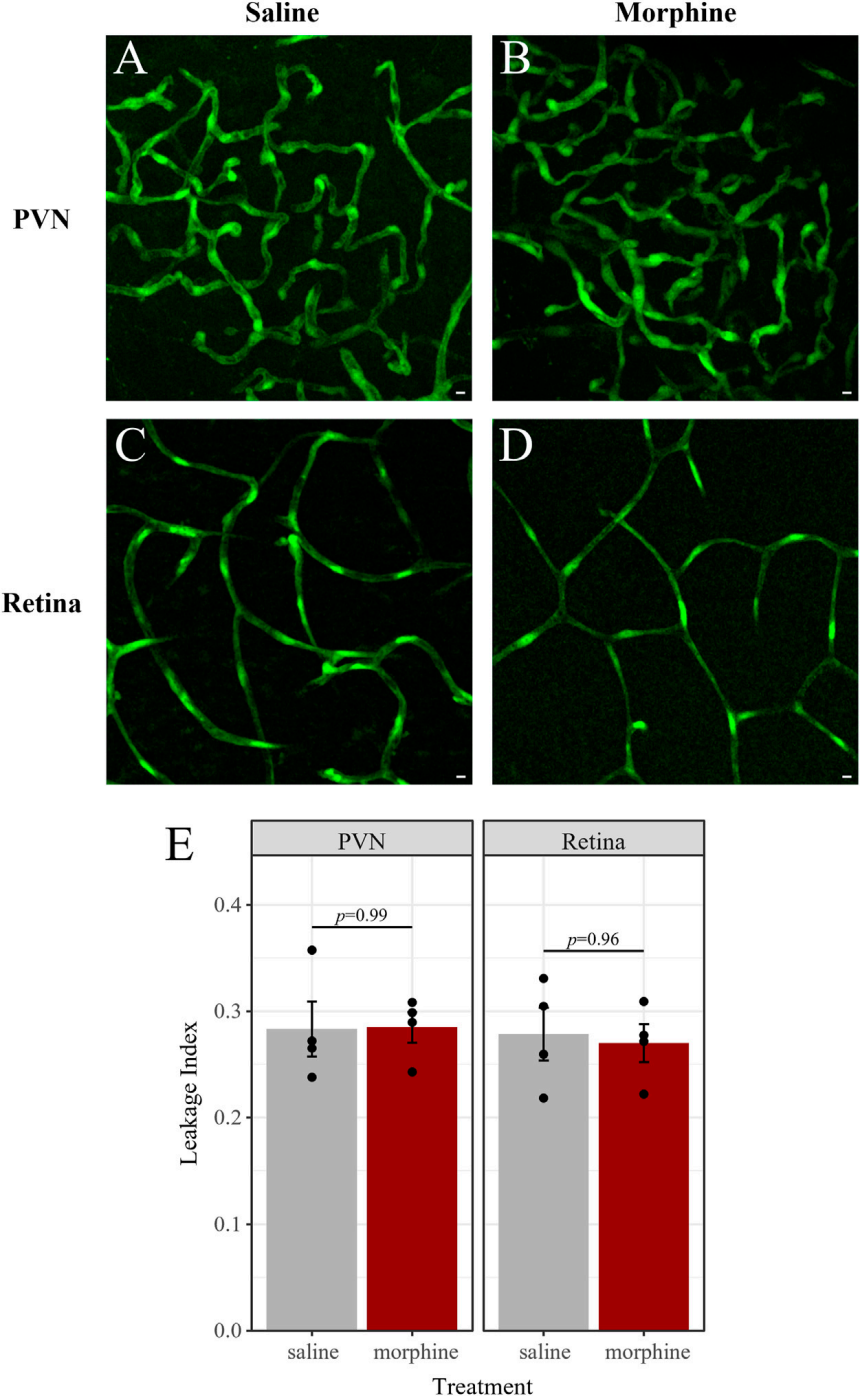
Chronic morphine treatment does not impact BBB or BRB permeability. Fluorescence imaging of FITC extravasation revealed no differences in barrier leakage between mice that received chronic morphine treatment (20 mg/kg, twice daily, 6 d; *n* = 4; **(B,D)** and vehicle-treated mice (saline, twice daily, 6 d; *n* = 4; **(A,C)**. Leakage was quantified in the densely vascularized PVN **(A,B)** and in retinal capillaries **(C,D)** of the same mice. **(E)** Leakage index is defined as the average intensity outside of vessels divided by the average intensity inside vessels. Data are presented as the mean ± SEM. Two-way ANOVA with Tukey *post hoc* adjustments found no significant differences (*p* < 0.05).

There is some evidence that chronic morphine exposure may impair the integrity of the BBB, which could affect morphine pharmacokinetics in the CNS (Leibrand et al., 2019). If our morphine treatment paradigm affects the integrity of blood-CNS barriers, it may increase the permeability of the barriers to xenobiotics such as morphine. To test this, we used fluorescence imaging of fluorescein isothiocyanate (FITC) extravasation to assess the integrity/permeability of the BBB and BRB (Figure 11). Six male mice were perfused with FITC, and an additional five male mice were perfused with FITC conjugated to 10 kDa dextran (FITC-dextran) (Supplementary Figure S3). In each group, mice received either chronic morphine treatment, vehicle (saline) or were left untreated. Treated mice were sacrificed 1 h after the last injection when morphine concentrations in the brain and retina are at their peak. Barrier leakage was assessed in the retina and the paraventricular nucleus of the hypothalamus (PVN) of each mouse. Notably, the PVN was chosen due to the dense vascularization found in this hypothalamic nucleus (Frahm and Tobet, 2015). Retinal arterioles in the superficial vascular layer, as well as capillaries in the superficial, intermediate, and deep layers of the retinal vasculature were imaged (Kornfield and Newman, 2014). Leakage indices were similar across these layers, thus capillaries in the intermediate layer were chosen as representative images (Figure 11; Supplementary Figure S3). Images of FITC extravasation of the retinal arterioles can be found in Supplementary Figure S4.

We obtained similar leakage indices (as defined in the Methods) in mice perfused with FITC and those perfused with FITC-dextran (Supplementary Figure S3). The average leakage index in each tissue and treatment combination was compared by ANOVA. We found that neither tissue (F (16) = 0.24, *p* = 0.63), treatment (F (16) = 0.14, *p* = 0.87), nor the interaction of tissue and treatment (F (16) = 0.95, *p* = 0.41) were significant predictors of the leakage index. Accordingly, there were no significant pairwise differences in the PVN or retina of saline- and morphine-treated mice (Figure 11), nor were these different from untreated mice (Supplementary Figure S3). There were also no significant differences between leakage indices in the PVN and retina of mice in the saline-treated group (t (8) = 0.16, *p* = 0.88), the morphine-treated group (t (8) = 0.47, *p* = 0.65), nor in the untreated group (t (8) = -1.3, *p* = 0.22). Thus, neither chronic morphine nor vehicle treatment significantly altered the permeability of the BBB or BRB.

## 3 Discussion

Previous work from our group showed that, in male mice, morphine deposition in the retina is higher than in the brain (Bergum et al., 2022a). Furthermore, we showed that morphine persists in the retina while it is cleared from both the systemic circulation and the brain (Bergum et al., 2022a). Thus, in this study we aimed to develop a mechanistic understanding of increased morphine penetrance and persistence in the retina compared to the brain, as well as to assess the role of sex/sex hormones in retinal morphine pharmacokinetics.

Here, we evaluated three ABC transporters (P-gp, Bcrp and Mrp2) as putative opioid transporters at the iBRB. We first validated the presence of these transporters within the retinal vasculature of the iBRB by immunohistochemistry. We found extensive P-gp and Bcrp labeling at the iBRB of both male and female mice, which is consistent with previous findings in male mice (Tagami et al., 2009; Chapy et al., 2016). Our analyses revealed that P-gp was exclusively expressed within occludin-labeled blood vessels. Interestingly, P-gp labeling was more robust in cryosectioned retinas than in whole-mount preparations (Figure 2; Supplementary Figure S1), however, we found true colocalization using Costes’ methods in all cases. This phenomenon may represent a decreased ability of the antibody to penetrate and bind P-gp in the whole-mount retina and/or a biologically significant clustering of P-gp at particular regions of iBRB vasculature that is more obvious in a whole-mount preparation. Bcrp immunolabeling was also primarily found in retinal blood vessels, however, some other retinal structures were labeled, which has not been previously reported (Tagami et al., 2009; Chapy et al., 2016). The robust expression of P-gp and Bcrp at the iBRB suggests that these transporters could contribute to morphine transport across the BRB in both males and females, as we did not uncover any differences between sexes in the immunolabeling for either transporter (Supplementary Figure S2). Conversely, we found that Mrp2 is not robustly expressed within blood vessels at the iBRB, which is consistent with previous reports that assessed other Mrp transporters (Tagami et al., 2009; Chapy et al., 2016). Its expression was more prevalent in cells near blood vessels, which may be neurons or cells that support the iBRB (e.g., pericytes, Müller glia). Although future studies could interrogate the identities of Mrp2-expressing cells in the retina, our results suggest that Mrp2 is not a prominent opioid transporter at the iBRB; it may instead play a supporting role in the regulation of drug transport between the systemic circulation and the retina. Furthermore, occludin expression is specific to the tight junctions between the endothelial cells of mammalian retinal vessels, but it is present in all the blood vessels of both the iBRB and outer BRB (Morcos et al., 2001). Thus, colocalization studies of P-gp and Bcrp using markers that are specific to the iBRB in adult mice (i.e., claudin-5) (Kojima et al., 2002; Greene et al., 2019) and/or that label, not just tight junctions, but the entirety of blood vessels (i.e., collagen IV) (Ramos et al., 2013) may provide additional insight into the expression and function of these transporters at the BRB.

After establishing that Bcrp and P-gp are robustly expressed at the iBRB, we wanted to determine the relative contribution of each protein to retinal morphine transport. Previous studies from our lab showed that P-gp expression is inversely correlated with morphine deposition when comparing the brain and retina (Bergum et al., 2022a); however, these studies were only performed in male mice. To establish a connection between morphine transporters at the iBRB and the potential role of sex/sex hormones in morphine pharmacokinetics, we performed experiments in male and female mice. Importantly, littermate female mice were divided into two different groups based on their estrus stage on the day of sacrifice. The first group of females was either in diestrus or metestrus (Low Estrogen/Progesterone ratio), with hormone profiles more similar to male mice. The second group of females was sacrificed during estrogen-dominant stages (High E/P), proestrus or estrus (Broestl et al., 2018). High E/P female mice have been shown to have lower sensitivity to tactile abdominal stimulation than low E/P females and male mice (Gonzalez and Carrasquillo, 2019; Tramullas et al., 2021). While assaying females in all four estrus stages would be ideal, these groupings were sufficient to elucidate the main effects of sex hormones. Furthermore, this would necessitate the use of at least twice as many female animals to reach the same end. Indeed, the lack of differences found in our study suggests further separation may not have provided more information. This work represents, to our knowledge, the first characterization of P-gp and Bcrp mRNA and protein expression in the male and female retina in the context of morphine treatment. Importantly, there is evidence of circadian oscillations in P-gp-mediated transport (Kervezee et al., 2014; Zhang et al., 2021), which may alter morphine pharmacokinetics over the course of the day. Since this study examined morphine pharmacokinetics and transporter expression at ZT 1, future experiments are needed to assess how circadian oscillations in transporter expression/function might alter morphine uptake and clearance from the CNS. Furthermore, future studies should also assess how circadian oscillations in opioid receptor expression (Naber et al., 1981; Mitchell et al., 1998; Jabourian et al., 2005; Yoshida et al., 2005; Takada et al., 2013) contribute to the behavioral effects of morphine and its deposition in the brain and retina.

We recapitulated findings that sex hormones influence P-gp and Bcrp expression in the brain, with expression being higher in High E/P females than in males. This is consistent with previous reports of testosterone inhibiting P-gp expression and efflux activity in tissues like the liver, kidney, and cornea (Dey et al., 2004; Suzuki et al., 2006; Shchul’kin et al., 2017), as well as reports that Bcrp is more highly expressed in the female rat brain than in males (Tanaka et al., 2005). Conversely, we did not find an effect of sex or estrus stage on the expression of either P-gp or Bcrp mRNA in the retina (Figure 8), consistent with the P-gp and Bcrp immunohistochemical labeling (Supplementary Figure S2). While we anticipated that circulating levels of sex hormones could influence opioid transporter expression, our work is consistent with their effect varying by tissue type and species (Dalla et al., 2022). Furthermore, we found no significant differences in morphine deposition in the brain or retina of males and females. This finding is consistent with past literature that examined morphine pharmacokinetics in the brain (Cicero et al., 1997) and plasma (Sarton et al., 2000), as well as a previous report that the effect of P-gp on morphine content in the brain is not different between female and male mice (Dagenais et al., 2001). In addition, these findings imply that the metabolism of the intraperitoneal morphine injections are not significantly impacted by sex hormones. While we cannot exclude the possibility that the pharmacokinetics of morphine shows sex-dependent differences at later time-points, our overall results suggest morphine would also accumulate in female retinas upon chronic administration, as previously shown for males (Bergum et al., 2022a). Intraperitoneal injections undergo rapid first-pass metabolism in the liver before reaching the systemic circulation (Handal et al., 2002; Turner et al., 2011), thus future studies using intravenous drug infusions might provide a clearer picture of the role that steroid hormones play in the regulation of opioid transport at the BBB and BRB.

After performing an extensive analysis of the expression patterns of P-gp and Bcrp in the CNS (brain/retina) of male and female mice, we wanted to weigh the relative contribution of each transporter to morphine deposition in the retina relative to the brain. We found that P-gp mRNA expression in the brain was nearly three-fold higher than Bcrp mRNA expression, which aligns with a report of higher expression of P-gp than Bcrp at the BBB measured by mass spectrometry (Kamiie et al., 2008). These data support the existing notion that P-gp is the primary opioid transporter at the BBB (Yang et al., 2018). Contrastingly, P-gp and Bcrp in the retina were similarly expressed in both males and females, with Bcrp expression being only slightly higher than P-gp in High E/P females. Despite their similar expression levels in the retina, two pieces of data suggest that P-gp is a more significant opioid transporter at the iBRB than Bcrp. First is P-gp’s exclusive presence in the iBRB compared to the expression of Bcrp by some other retinal structures. Second, only P-gp expression significantly correlated with morphine deposition in the retina, whereas the relationship between retinal Bcrp and morphine content was not statistically significant. Interestingly, the expression levels of both transporters appeared to be relatively poor predictors of brain morphine deposition, suggesting that neither P-gp nor Bcrp expression directly affect morphine concentration in the brain. Although our data indicates that P-gp is the main transporter involved in regulating morphine deposition in the retina, there is evidence that suggests P-gp and Bcrp work cooperatively to efflux common substrates at the BBB (Agarwal and Elmquist, 2012; Yang et al., 2018). At the iBRB, it has been shown that P-gp transport of verapamil is greater than that by Bcrp (Chapy et al., 2016), however, transporter function is known to be substrate dependent (Yang et al., 2018). Thus, functional studies of opioid (e.g., morphine) transport by P-gp and Bcrp at the iBRB are needed to elucidate the contributions and potential cooperative effect of these transporters. Such investigations should include studies that test the role of these ABC transporters in the retinal deposition of morphine, as well as other commonly administered opioids that are known P-gp substrates, such as methadone, buprenorphine, and fentanyl (Yang et al., 2018).

At first glance, the finding that P-gp expression in the retina, but not the brain, correlates positively with CNS morphine concentrations appears to be at odds with previous findings that P-gp negatively correlates with morphine deposition (Hamabe et al., 2007). Indeed our previous paper reported an inverse relationship between P-gp expression and morphine deposition when comparing the brain and retina (Bergum et al., 2022a). However, we do not believe that these findings are necessarily inconsistent with previous studies examining P-gp’s role in morphine transport across blood-CNS barriers. The positive relationship between P-gp expression at the iBRB and morphine deposition in the retina may be due to the relatively low abundance of this transporter at this barrier. In other words, P-gp expression in the brain is much more robust; therefore, small reductions in P-gp expression will not significantly alter the morphine transport across the BBB. On the other hand, with relatively low P-gp expression in the retina, every P-gp transporter at the iBRB has a larger effect on the overall morphine transport. The interpretation of this data is particularly difficult when we consider that P-gp could be expressed at both the basal and laminar sides of the endothelial cells that make up the iBRB and BBB (Yang et al., 2018). While ABC transporters are typically believed to be located on the luminal membrane of capillary endothelial cells to facilitate morphine efflux (Viscusi and Viscusi, 2020), localization to the sublaminar/basolateral membrane could also facilitate morphine transport into the CNS (Yang et al., 2018). High resolution imaging along with functional transport studies may provide a more detailed understanding of the role of P-gp in the influx/efflux of drugs like morphine across blood-CNS barriers.

Opioid pharmacokinetics in the CNS may not only depend on the activity of transporter proteins but on the integrity of blood-CNS barriers. Fluorescence imaging of FITC or FITC-dextran extravasation is a common assay for measuring BBB or BRB permeability (Li et al., 2011; Miyata and Morita, 2011; Liang et al., 2012; Frahm and Tobet, 2015; Natarajan et al., 2017; Xu et al., 2019). There have been reports of morphine exposure impairing barrier integrity *in vitro* through a reduction of tight junction protein ZO-1 (Wen et al., 2011) as well as reports that chronic morphine exposure (via time-released pelleted implants) increases BBB permeability to fluorescently-labeled dextrans in mice (Leibrand et al., 2019). However, it has also been suggested that, rather than chronic morphine exposure itself affecting BBB permeability, withdrawal from the drug increases BBB permeability (Sharma and Ali, 2006). This is similar to the previous uncertainty regarding whether chronic morphine exposure caused an upregulation of P-gp expression, which is now thought to be a withdrawal effect (Yousif et al., 2008; Yousif et al., 2012; Bergum et al., 2022a). Given that morphine clears from the brain within just 3 h post-injection (Bergum et al., 2022a), it is critical to harvest brain tissue soon after the drug administration to differentiate the effects of morphine exposure from withdrawal effects. As such, we performed these assays approximately 1 h after the injection to ensure peak concentrations of morphine were present in the brain and retina. We found no effect of chronic morphine treatment on barrier permeability, suggesting that BRB integrity does not contribute to the selective accumulation of morphine in the retina. Although there is evidence that sex hormones (as well as age and disease) can influence blood-CNS barrier integrity (Bake and Sohrabji, 2004; Wilson et al., 2008; Cipolla et al., 2009; Atallah et al., 2017; Dion-Albert et al., 2022), the permeability of the BBB was reported to be similar in male and female healthy adult mice (Schaffenrath et al., 2021). In conjunction with the lack of sex differences in morphine pharmacokinetics and transporter expression in the retina presented here, these data suggest that barrier integrity does not influence morphine pharmacokinetics and is therefore not a likely source of the sex-dependent differences in morphine efficacy.

The current study establishes the role of ABC transporters in morphine’s selective persistence and accumulation in the eye: a phenomenon that has been shown not just in mice (Bergum et al., 2022a) but in humans as well (Wyman and Bultman, 2004; Bévalot et al., 2016). We found that morphine deposition in the CNS (retina/brain) is not dependent on sex/sex hormones. While this is consistent with previous reports, questions remain as to the molecular origins of sex differences in the efficacy of and outcomes associated with opioid drugs. The role of sex on opioid-mediated antinociception is complex, ranging from the physiological (i.e., sex hormones, neuroanatomical differences) to the psychosocial (Nasser and Afify, 2019). As with opioid-mediated antinociception, the relationship between opioids, sleep/wake disturbances and sex is still being unraveled (Tripathi et al., 2020; Huhn and Finan, 2021). Some evidence suggests women are more likely to experience opioid-induced sleep disturbances (OISD) (Finan et al., 2020; Ellis et al., 2022). Importantly, OISD are emerging as a promising therapeutic target for opioid use disorder (Huhn and Finan, 2021). While the mechanisms underlying OISD are still not known, our recent work suggests that opioids modulate sleep/wake behavior through the μ-opioid receptors (MORs) expressed by intrinsically photosensitive retinal ganglion cells (ipRGCs) (Tripathi et al., 2020; Huhn and Finan, 2021; Bergum et al., 2022c). Indeed, endogenous opioid peptides activate the MORs expressed by ipRGCs to modulate healthy sleep/wake (Berezin et al., 2022). Furthermore, the MORs expressed by ipRGCs mediate morphine-induced sleep/wake changes (i.e., behavioral sensitization) (Bergum et al., 2022b). Thus, the persistence of morphine in the retina may allow for prolonged activation of the MORs expressed by ipRGCs, which could disrupt endogenous opioid signaling and lead to OISD (Bergum et al., 2022b; Berezin et al., 2022; Bergum et al., 2022c). The results presented here position P-glycoprotein expression as an important determinant of retinal morphine pharmacokinetics in the inner retina. Continued characterization of opioid pharmacokinetics in the brain and retina will facilitate the development of potential therapeutics for OISD and opioid use disorder.

## 4 Materials and methods

### 4.1 Animals

Adult male and female mice (8–26 weeks) were kept on a 12-h light:12-h dark cycle with lights on at 7:00 a.m. (Zeitgeber time ZT 0), fed standard chow and water *ad libitum*. All animal protocols were approved by the Colorado State University Institutional Animal Care and Use Committee in accordance with the NIH Guide for the Care and Use of Laboratory Animals.

Mice with Cre recombinase expressed upstream of the melanopsin (*Opn4*) promoter [Tg (*Opn4*-cre)SA9Gsat/Mmucd, #036544-UCD; *Opn4*-cre] were purchased from the Mutant Mouse Resource and Research Center (MMRRC) at the University of California at Davis. *Opn4*-cre mice were backcrossed into a 100% C57BL/6J background prior to purchase and maintained as hemizygotes (*Opn4*-cre +/−).

McKO mice, in which only ipRGCs lack functional MORs, were generated as described previously (Weibel et al., 2013; Cleymaet et al., 2021; Bergum et al., 2022b; Berezin et al., 2022). *Oprm1* ^fl/fl^ breeders (Jackson Labs strain #030074) were generously provided by Dr. Brigitte Kieffer (Douglas Research Center, McGill University). *Oprm1* ^fl/fl^ mice have exons 2 and 3 of the μ-opioid receptor (*Oprm1*) gene flanked by loxP sites and were maintained on a 50% C57BL/6J-50% 129Sv background for 5 generations before receipt (Weibel et al., 2013). *Oprm1* ^fl/fl^ and *Opn4*-cre mice were crossed to produce 50% *Oprm1* ^fl/fl^ mice and 50% McKO mice with the floxed *Oprm1* gene on both alleles and *Opn4*-cre on one allele. This Mu^fl^/McKO line was maintained on a background containing at least 75% C57BL/6J and 25% 129Sv. Male and female McKO mice, and their littermate controls, were used for the mass spectrometry and qRT-PCR experiments in the context of morphine treatment. Mice of this mixed background have been used in previously published studies on retinal morphine pharmacokinetics, and the pharmacokinetics of morphine in the retina and the brain were not found to be significantly different between McKO mice and their wild-type (C57BL/6J) counterparts (Bergum et al., 2022b; Bergum et al., 2022b).

Mice carrying a bacterial artificial chromosome (BAC) in which the melanopsin (*Opn4*) promoter drives expression of enhanced green fluorescent protein (EGFP) [Tg (Opn4-EGFP)ND100Gsat/Mmucd] were obtained from the Gene Expression Nervous System Atlas (GENSAT) project. These mice are hereafter referred to as *Opn4*:EGFP mice and were maintained on a C57BL/6J background. Untreated male and female *Opn4*:EGFP and *Opn4*-cre mice, and their littermate controls, were used for immunohistochemical and quantitative reverse transcription PCR (qRT-PCR) experiments, as both strains are on the commonly used C57BL/6J background.

*Opn4*-cre mice were crossed to Ai14 (Jackson Labs strain #007914) mice to express tdTomato following *Opn4*-driven Cre-dependent recombination (*Opn4*-cre-tdTomato). *Opn4*-cre-tdTomato mice were on a 100% C57BL/6J background. Male *Opn4*-cre-tdTomato mice, and their littermate controls, were used in the fluorescence extravasation studies. Positive *Opn4*-cre-tdTomato expression in the ganglion cell layer was used to guide the imaging of distinct retinal vasculature layers and did not affect FITC extravasation.

#### 4.1.1 Estrous staging and grouping

Vaginal lavage was performed on group-housed female mice each morning for at least one estrus cycle (5 + d). The stage of estrus was determined by cytological evaluation as previously described (McLean et al., 2012). We aimed to group female mice into two groups based on their hormone profiles: females in proestrus and estrus, in which the ratio of estrogen to progesterone is relatively high (High E/P); and females in metestrus and diestrus, in which the estrogen to progesterone ratio is lower (Low E/P). As mice were regularly cycling, the day of sacrifice was determined by the predicted stage(s) needed to secure a large enough sample size in the Low E/P and High E/P groups. Based on our previous qRT-PCR experiments measuring P-gp expression, our target sample size was 5 to achieve 0.95 power to detect a 1.5-fold change with a standard deviation of 0.5.

#### 4.1.2 Morphine treatment

Mice were weighed to ensure proper dosing. For acute treatment, mice were injected with a single i.p. dose of 20 mg/kg morphine (Morphine sulfate salt pentahydrate, Sigma-Aldrich Saint Louis, MO, United States; Product Number: M8777, dissolved in sterile saline) at light onset (ZT 0) as previously described (Bergum et al., 2022a). The chronic morphine treatment paradigm consisted of 5 days of twice daily 20 mg/kg morphine (administered at ZT 0 and ZT 12), followed by a final 20 mg/kg i.p. injection at ZT 0 on day 6 of the treatment paradigm (Bergum et al., 2022a).

### 4.2 Morphine analysis by liquid chromatography tandem-mass spectrometry

#### 4.2.1 Tissue sample collection: retina, brain, and plasma

Mice were deeply anesthetized with Fluriso (isoflurane, VetOne), decapitated, and trunk blood was collected into EDTA-coated tubes (BD Vacutainer K2 EDTA 7.2 mg tube 4.0 mL). Then, the cortex was removed, weighed, and placed in tubes containing 100 µL 0.1 M PBS. Whole retinas were then microdissected from the eyes of each animal and placed in tubes containing 100 µL 0.1 M PBS. Blood was centrifuged and the plasma, brain and retina were stored at −20°C.

#### 4.2.2 Retina batch preparation

We prepared retina samples from analysis as previously described (Bergum et al., 2022a). In short, to prepare retina samples for analysis, 10 µL of 10 μg/mL D6-morphine internal standard solution (Morphine-D6 solution, Cerilliant, Round Rock, TX, United States) was added to whole-retina samples and matrix-matched standards. Subsequently, 200 µL ice-cold acetonitrile was added to the retina samples/standards and the samples/standards were sonicated at maximum power (40 kHz) for 30 min using a Branson Bransonic^®^ 5800 Ultrasonic bath (Emerson Electric Co. Saint Louis, MO, United States). Retina samples were then spun down at 14,000 rpm for 5 min, and 100 µL supernatant was added to sample vials containing 200 µL 0.1% formic acid in water and then transferred to autosampler vials fitted with 400 μL glass inserts. The limit of detection was 20 ng/mL morphine.

#### 4.2.3 Brain batch preparation

We prepared brain samples from analysis as previously described (Bergum et al., 2022a). To prepare brain samples for analysis, 10 µL of 1 μg/mL D6-morphine internal standard solution was added to brain samples and matrix-matched standards. Next, brain samples/standards were then homogenized for 10 s using a BeadBug™ 3 Position Bead Microtube homogenizer (Benchmark Scientific, Inc. Sayreville, NJ, United States) in 400 µL ice-cold acetonitrile. Samples were then centrifuged 14,000 rpm for 5 min and the supernatant was added to Phenomenex Strata-X-Drug B solid phase columns. Columns were washed with 2 mL 0.1% formic acid in water and 2 mL methanol. The columns were then dried for 10 min prior to elution. Two successive aliquots of a solution containing 50% acetonitrile, 42% methanol, and 8% 7 N ammonium in methanol were used to elute samples from the columns. Eluents were collected into a clean glass test tube and dried under nitrogen at 40°C. Dried eluents were reconstituted with 200 µL 95:5% water:acetonitrile and transferred to autosampler vials fitted with 400 μL glass inserts. The limit of detection was 0.2 ng/mL morphine.

#### 4.2.4 Plasma batch preparation

We prepared plasma samples from analysis as previously described (Bergum et al., 2022a). To prepare serum samples for analysis, 10 µL of 1 μg/mL D6-morphine internal standard solution was added to serum samples and matrix-matched standards. Serum samples/standards were prepared for solid phase extraction by adding 20 µL zinc sulfate (5% weight/volume), followed by the addition of 300 µL ice-cold acetonitrile to each tube. Samples were then centrifuged at 14,000 rpm for 5 min and supernatant was added to Phenomenex Strata-X-Drug B solid phase columns. Columns were washed with 2 mL 0.1% formic acid in water and 2 mL methanol. The columns were then dried for 10 min prior to elution. Two successive aliquots of a solution containing 50% acetonitrile, 42% methanol, and 8% 7 N ammonium in methanol were used to elute samples from the columns. Eluents were collected into a clean glass test tube and dried under nitrogen at 40°C. Dried eluents were reconstituted with 200 µL 95:5% water: acetonitrile and transferred to autosampler vials fitted with 400 μL glass inserts. The limit of detection was 0.5 ng/mL morphine.

#### 4.2.5 Data acquisition and analysis

Samples were analyzed with an Agilent 1290 UHPLC coupled to an Agilent 6460 triple quadrupole mass spectrometer equipped with an Agilent Jet Stream electrospray ionization. Morphine was chromatographically separated on a Restek Raptor biphenyl column (3.0 × 50 mm, 2.7 μm) held at 40°C. A sample volume of 10 μL was injected into a mobile phase mixture of 95% water with 0.1% formic acid (A) and acetonitrile with 0.1% formic acid (B). The flow rate of 0.4 mL/min was kept consistent throughout the run time. The mobile phase composition was held at 3% B for 1.5 min, increased to 40% B at 3 min, and finished at 100% B at 4 min. The ionization source conditions used were as follows: positive polarity and nebulizer 40 psi; gas flow of 10 L/min at 320°C; sheath gas flow of 12 L/min at 380°C; capillary voltage of 3750 V; and nozzle voltage of 500 V. The ion transitions monitored were 286.2- > 152 and 128 m/z for morphine and 292.2- > 152 and 128 m/z for D6-morphine. Analytes were confirmed by retention time and the product ion ratio correlation between the sample peaks and corresponding standards (±20%). The data collection and processing were performed by using Agilent (Santa Clara, CA, United States) Mass Hunter Quantitative software (v.B.08.01). Quantitation was performed with linear regression using seven-point calibration curves from 0.5 ng/mL to 500 ng/mL (for brain and serum) or six-point calibration curves from 5 ng/mL to 1 μg/mL (for retina). These calibration curves were made from matrix (brain, serum, or retina tissue derived from untreated animals) spiked with morphine standards (Morphine solution, Sigma-Aldrich Saint Louis, MO, United States; Product Number: M-005).

### 4.3 Immunohistochemistry

Procedures were as previously described (Berezin et al., 2022). Mice were anesthetized with Fluriso (isoflurane, VetOne) and sacrificed by cervical dislocation. After enucleation, the eyes were dissected in 0.1 M phosphate buffered saline (PBS; pH 7.4) and fixed in freshly prepared 4% paraformaldehyde (PFA) in PBS. For cryosectioning, eyecups were immersed then in 30% sucrose at 4°C for several days prior to being embedded in Optimal Cutting Temperature (O.C.T.) Compound (Tissue-Tek). The tissue was sectioned at 20 μm on a ThermoFisher CryoStar NX50 cryostat, directly mounted on SuperFrost Plus slides (Fisher Scientific), and stored at −20°C until use. For whole-mount preparations, immunohistochemistry was performed immediately following fixation. Both tissue types were subject to antigen retrieval. As previously described (Gallagher et al., 2012), the tissue was incubated in boiling 10 mM sodium citrate in PBS for 15 min followed by incubation in 5% sodium borohydride in PBS for 45 min. The tissue was then incubated in a blocking solution (5% serum and 0.5% Triton X-100 in PBS) for 3 h on a shaker table at RT. Retinas were incubated in primary antibodies diluted in the blocking solution for 24–36 h on a shaker table at RT (rabbit anti-P-glycoprotein 1:200, Abcam [EPR10364-57] #ab170904; rat anti-Bcrp 1:50, Enzo [BXP-53] #ALX-801–036; rabbit anti-Mrp2 1:100, Bioss #bs-1092R; mouse anti-occludin 1:250, Invitrogen [OC-3F10] #33–1500). Sections were then washed in PBS and incubated, either at RT for 2 h or overnight at 4°C, in the appropriate secondary antibodies. After the final washes in PBS, retinas were stained with DAPI (Advanced Cell Diagnostics, Newark, CA) and mounted in Vectashield Plus Antifade Mounting Medium (Vector Laboratories, Burlingame, CA).

#### 4.3.1 Image analysis

All images were obtained on a Zeiss LSM 900 confocal microscope (Carl Zeiss, Oberkochen, Germany). For all acquisitions, sequential scans at the different wavelengths were performed. For immunohistochemical experiments performed in whole-mount retinas, four to five ∼320 μm^2^ areas of the peripheral retina were imaged. Z-stack images were taken at 1 μm increments, starting with the superficial plexus in the ganglion cell layer through the apparent end of the retinal vasculature (≥20 μm) with a ×20 air objective. For immunohistochemical experiments performed on cryosectioned retinas, five to six Z-stack images were taken per mouse at 1 μm increments throughout the thickness of sections (∼20 μm) with a ×20 air objective (image size ∼320 μm^2^) or ×40 oil-immersion objective (image size ∼160 μm^2^). Images were denoised using the Despeckle and Remove Outlier (6 pixel radius, threshold = 3) functions in Fiji (Schindelin et al., 2012). Colocalization of P-glycoprotein and occludin was determined using Manders’ coefficients (Manders et al., 1993) as well as Costes’ automatic threshold and randomization procedures (Costes et al., 2004) using the Coloc2 plugin in Fiji (Schindelin et al., 2012). True colocalization was found when Costes’ *p*-value was greater than 95% (Costes et al., 2004).

### 4.4 Quantitative reverse transcription PCR (qRT-PCR)

Procedures were performed as previously described (Bergum et al., 2022a; Berezin et al., 2022), with minor modifications described below.

#### 4.4.1 cDNA preparation

Mice received morphine or saline as described in Section 4.2.1. One hour after the (last) injection (ZT 1), the mice were anesthetized with isoflurane and sacrificed by decapitation. Hypothalamic tissue was dissected from the brain and immediately processed, while retinas were microdissected in 0.1 M phosphate buffered saline (PBS; pH 7.4) and placed in RNAlater solution (Sigma-Aldrich R0901) at 4°C until all samples were obtained. Total RNA was extracted from both tissues using the Extracta Plus RNA Kit (Quantabio, Beverly, MA), which contains a genomic DNA removal column, according to the manufacturer’s instructions. Briefly, the tissues were lysed in Buffer EPRL supplemented with DTT (GoldBio, St. Louis, MO, United States). Hypothalamic tissue was homogenized by passing it through a 20 g needle 5 times, followed by a 27 g needle 5 times, then frozen at −80°C until all samples were obtained. Retinal tissue was disrupted with a micropestle and homogenized by passing it through a 20 g needle 5 times. The lysates were centrifuged, and the supernatants combined with 70% ethanol before being put on the column. After RNA was eluted from the column, the concentration and quality (A260/A280) of each sample was assessed using a Nanodrop (ThermoFisher NanoDrop Lite, Waltham, MA, United States). RNA integrity was assessed on a 1% agarose gel. All samples displayed strong 28S and 18S rRNA bands, no gDNA contamination, and no degradation. From each sample, 200 ng RNA was reverse-transcribed into cDNA using qScript cDNA SuperMix (Quantabio, Beverly, MA) according to the manufacturer’s instructions. The cDNA was then stored at −20°C until it was used for qPCR amplification.

#### 4.4.2 Primer design

Primer sequences against rat *Abcb1a* (P-gp) were previously adapted for use in mouse (Yousif et al., 2008; Bergum et al., 2022a). Primers against *Abcg2* (Bcrp) were adapted from previously published primers used in rat (Yousif et al., 2012). Primers against *Abcc2* (Mrp2) were obtained from previously published sequences (Liu et al., 2018). The probe sequences for P-gp, Bcrp and Mrp2, as well as primer and probe sequences for the reference genes β-actin and Tbp, were designed using Integrated DNA Technologies’ PrimerQuest™ Tool (Bergum et al., 2022a; Berezin et al., 2022). β-actin is a commonly used reference gene that has been used successfully in mouse retina (Sawant et al., 2017), and Tbp has been shown to be an appropriate reference gene for adult mouse retina (Adachi et al., 2015). The primer and probe sequences can be found in Table 1.

**TABLE 1:** Primer and probe sequences used for qRT-PCR.

Target	Forward primer sequence (5′-3′)	Reverse primer sequence (5′-3′)	Probe sequence (5′-3′)
P-gp (*Abcb1a*), NM_011076.3	CAG​CCA​GCA​TTC​TCC​GTA​ATA	CCC​AAG​GAT​CAG​AAA​CAA​CA	/56-FAM/CAGCGGCAG/ZEN/AA CAGCAACTTGTTT/3IABkFQ/
Bcrp (*Abcg2*), NM_001381927.1	CAG​CAG​GTT​ACC​ACT​GTG​AG	CAT​CAC​AGC​AGA​AGA​ATC​TCC​A	/56-FAM/CCCTACAAC/ZEN/AA CCCTGCGGATTT/3IABkFQ/
Mrp2 (*Abcc2*), NM_013806.2	GGA​TAA​TGA​GGC​GCC​GTG​GGT	CCG​GCC​GAT​ACC​GCA​CTT​GA	/56-FAM/CCGACAAGA/ZEN/A GCCTCCGGCAGATT/3IABkFQ/
β-actin (*Actb*), NM_007393.5	GTC​ATC​CAT​GGC​GAA​CTG​G	ACTGTCGAGTCGCGTCC	/5HEX/CGTTGCCGG/ZEN/TCCACACCCGCCA/3IABkFQ/
TATA box binding protein (*Tbp*), NM_013684.3	CCA​TGA​AAT​AGT​GAT​GCT​GGG​C	GGG​TAT​CTG​CTG​GCG​GTT​T	/5HEX/TGCGGTCGC/ZEN/GTCATTTTCTCCGCAGT/3IABkFQ/

For primer design using the Integrated DNA Technologies’ PrimerQuest™ Tool, primers were designed to span an exon–exon junction, have melting temperatures of between 60°C and 65°C, have a GC content of between 45% and 65%, and be no more than 30 nucleotides long. Probes were designed to have melting temperatures at least 5°C higher than the primers, have a GC content of between 45% and 75%, and be no more than 30 nucleotides long. Amplicons were required to be shorter than 150 nucleotides. All other parameters were left as defaults.

Upon receipt, the multiplexed primers (500 nM) and probe (250 nM) were resuspended in an IDTE buffer (10 mM Tris, 0.1 mM EDTA in H2O) upon receipt, then stored at −20°C. PCR products from each primer set were run on a 2% agarose gel to assess the specificity of the primers. For all genes, one clear band was seen with no gDNA contamination or additional products. The reaction parameters were first determined in singleplex reactions before multiplexing. For each primer set, a temperature gradient was used to determine the optimal annealing temperature.

#### 4.4.3 qRT-PCR protocol and analysis

Reactions were set up using GoTaq^®^ Probe qPCR Master Mix (ProMega) according to the manufacturer’s instructions; however, because our primers and probes arrived multiplexed, only 1 µL total was used, and an additional 2 µL of nuclease-free water was added to the total 20 µL reaction mix. The cycling conditions were as follows: 2 min, 95°C, GoTaq^®^ DNA polymerase activation, and then 35 cycles of denaturation (95°C, 15 s) and annealing/extension (63°C, 30 s).

Each plate contained a standard curve run in triplicate, which included three 10-fold dilutions. Every experimental sample was run in triplicate using 2 µL of cDNA. Both reference genes were stably expressed in all samples. In each run, a “no reverse-transcription” control (RNA that was not reverse-transcribed) and no-template control (H_2_O instead of cDNA or RNA) was included in each run. No amplification was seen in any negative controls. Assay plates were run on a CFX96 Touch Real-Time PCR Detection System (BioRad, Hercules, CA, United States).

The CFX Manager™ Software Version 3.1 (BioRad, Hercules, CA, United States) was used to set the threshold for each reaction (the highest *R* ^2^ just above the background) and to assess the efficiency of the reaction based on the standard curve (90%–110%). It was also used to normalize the data between plates when samples needed to be re-run or run on multiple plates. In these cases, at least one identical sample was run on each plate and used as an inter-run calibrator. The Cq values for each experiment were downloaded as an Excel spreadsheet and the ΔΔCt method was used for analysis (Livak and Schmittgen, 2001; Hellemans et al., 2007). For each gene, the Cq values for all samples in the control group were averaged and used as the reference to calculate the relative gene expression (RGE) in each sample. Because multiple reference genes were used, the geometric mean of those genes’ relative quantities was used to calculate the RGE (Vandesompele et al., 2002).

### 4.5 Fluorescence extravasation assays

Male mice received i.p. injections of chronic morphine or vehicle (saline) as described in Section 4.2.1. One hour following the last injection, mice were deeply anesthetized with Fluriso (isoflurane, VetOne) and kept anesthetized by continuous isoflurane delivery via a nose-cone. Mice were transcardially perfused with 10 mL of FITC (fluorescein isothiocyanate, 1 mg/mL, ThermoScientific #46424) or FITC-Dextran (fluorescein isothiocyanate dextran 10 kDa, 0.5 mg/mL Fisher Scientific #F0918100MG) dissolved in PBS (0.1 mg/mL) at a rate of 4 mL/min using a syringe pump (New Era Pump Systems NE-300). Mice were then perfused with 15 mL of 4% PFA in PBS. The eyes were enucleated and post-fixed in 4% PFA at RT for 25 min (20 min puncture-fix, retinas dissected, 5 min fixation). Retinas were washed in PBS and then mounted in Vectashield Plus Antifade Mounting Medium (Vector Laboratories, Burlingame, CA). Whole brains were removed and post-fixed in 4% PFA at 4°C for at least 24 h and then cut into 100 μm coronal sections on a Leica vibratome (VT1200S).

To evaluate the permeability of the blood-brain barrier, we assessed the densely vascularized paraventricular nucleus of the hypothalamus (PVN). Z-stacks of PVN vasculature (≥20 μm) were taken in 1 μm increments on either side of the third ventricle. To evaluate the permeability of the blood-retina barrier, we obtained one to two representative Z-stack images of the central arterioles and venules and one to two representative Z-stack images of the capillary vasculature of the peripheral retina (all in 1 μm increments). Retinal capillaries were imaged from the superficial plexus in the ganglion cell layer through the apparent end of the retinal vasculature (≥20 μm). All images were ∼320 μm^2^ and obtained with a ×20 air objective on a Zeiss LSM 900 confocal microscope (Carl Zeiss, Oberkochen, Germany).

Images were denoised using rolling ball background subtraction (25 pixels) followed by the Despeckle and Remove Outlier (3 pixel radius, threshold = 10) functions in Fiji (Schindelin et al., 2012). Maximum intensity Z-stack projections were created from each image (retinal capillary images were split into two stacks: one from the superficial plexus to the intermediate plexus, and the second from the intermediate plexus to the deep plexus terminating in the outer plexiform layer). Each maximum projection image is composed of 5–10 single optical sections taken at 1 μm increments, and thus represents approximately 5–10 μm of tissue vasculature. Images were thresholded using the Li method in Fiji and converted to binary masks. A composite image of each mask and the denoised image were visually inspected to ensure accurate representation of the imaged vasculature. Barrier leakage was quantified as previously described (Frahm and Tobet, 2015). The mask containing vessel “objects” was redirected to the denoised image and the average intensity within each vessel was measured. Each vessel “object” was expanded by 2 μm and the region outside each vessel was saved; the average intensity in each outside region was measured. Leakage index was defined as the average intensity outside the vessel divided by the average intensity inside the vessel. The final reported leakage index is the average across all vessels in all images of the PVN or retina of each mouse.

In order to determine whether FITC type should be included as a factor in our statistical model, we compared two mixed models of the data by ANOVA: 1) with tissue and treatment as dependent variables, and 2) with tissue, treatment, and FITC type as dependent variables. Both models had leakage index as the independent variable and had samples identified by mouse ID number. We found that the more complex model (2) was not significantly better than model (1) without FITC type (*p* = 0.223): the simpler model (1) had a lower Akaike Information Criterion (AIC; −65.8 compared to −62.1) and Bayes Information Criterion (BIC; −57.1 compared to-46.8).

### 4.6 Statistical analysis

All data processing, visualizations and statistical tests were performed using R (v4.2.1; R Core Team, 2022) in RStudio (v2022.7.2.576; R Studio Team, 2022). Data visualizations were made using the *ggplot2* package in R (Wickham, 2016). For all tests, *p* < 0.05 was considered significant. T-tests and ANOVAs with *post hoc* Tukey adjustments were used when data were normally distributed and (if applicable) had homogeneous variance across groups. Whether the data was transformed (e.g., log_2_ or ln) to satisfy these assumptions is noted in the text. Linear mixed-effects models were fit using the *lme4* package in R (Bates et al., 2015), and pairwise comparisons were calculated using the *emmeans* package in R (Lenth, 2022).

## Figures and Tables

**Table:** 

**ABC**	ATP-binding cassette transporter
**BBB**	Blood-brain barrier
**BRB**	Blood-retina barrier
**Bcrp**	Breast cancer resistance protein (*Abcg2*)
**CNS**	Central nervous system
**E/P**	Estrogen to progesterone ratio
**GCL**	Ganglion cell layer
**iBRB**	Inner blood-retina barrier
**INL**	Inner nuclear layer
**Mrp2**	Multidrug resistance protein 2 (*Abcc2*)
**ONL**	Outer nuclear layer
**OPL**	Outer plexiform layer
**P-gp**	Permeability glycoprotein (*Mdr1a*/*Abcb1a*)
**PR**	Photoreceptor layer
**PVN**	Paraventricular nucleus of the hypothalamus
**RGE**	Relative gene expression

## Data Availability

The original contributions presented in the study are included in the article/Supplementary Material, further inquiries can be directed to the corresponding author. The data and the code used for analysis can be found at https://github.com/thebergular/morphine_sex_differences_brb_bbb.
