# Supplementary material for: Morphine pharmacokinetics and opioid transporter expression at the blood-retina barrier of male and female mice
Source: Front Pharmacol. 2023 Jun 14;14:1206104. doi: 10.3389/fphar.2023.1206104 (PMC10301758; doi:10.3389/fphar.2023.1206104)
Supplement: Supplementary file 1 [file DataSheet1.docx]

Supplementary Material

Morphine pharmacokinetics and opioid transporter expression at the blood-retina barrier of male and female mice

Casey-Tyler Berezin^†^, Nikolas Bergum^†^, Glenda M. Torres Lopez, Jozsef Vigh*

^†^These authors share first authorship.

*** Correspondence:** Jozsef Vigh: [jozsef.vigh@colostate.edu](mailto:jozsef.vigh@colostate.edu)

**
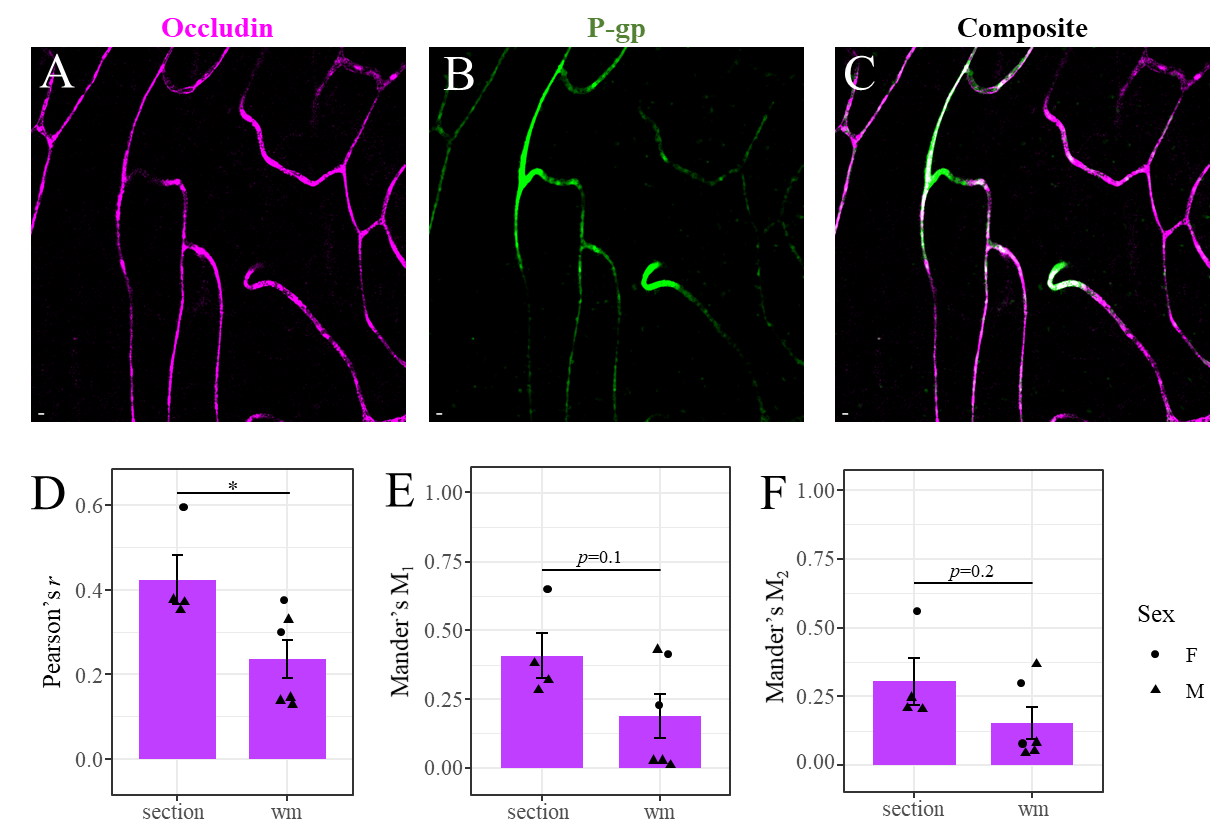
**

**Fig S1. P-gp expression is restricted to the iBRB in a whole mount retina.**

(**A-C**) Immunohistochemistry in male (triangles) and female (circles) whole-mount retinas reveals P-gp (**B**, green) labeling is restricted to the occludin-labeled iBRB (**A**, magenta [pseudocolored]), however the robustness of P-gp immunolabeling varies throughout the vasculature. P-gp positively colocalized with occludin in all images (Costes’ methods, P=100%; n=27 [4-5/mouse, 6 mice]). Image is a single optical section, scale bar = 5 μm. (**D-F**) Pearson’s correlation coefficient (*r*) and Mander’s M_1_ and M_2_ coefficients (indicating the extent of occludin-labeled area overlapping with P-gp, and vice versa); each point represents the average across all images obtained from a single mouse. Data presented as mean ± SEM. * *p*<0.05


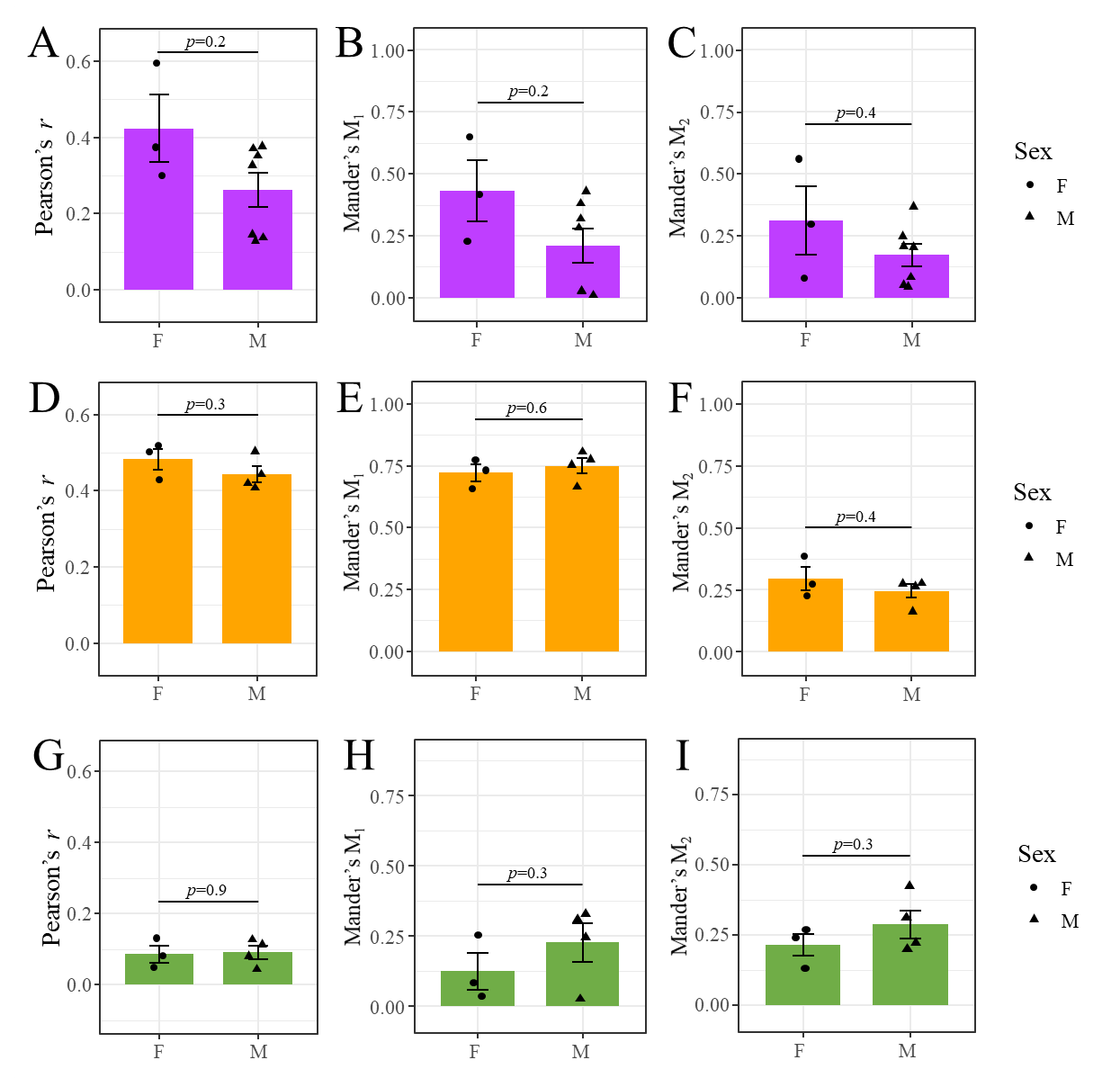


**Fig S2. Immunolabeling for P-gp, Bcrp and Mrp2 are not quantitatively different between male and female mice.**

Colocalization parameters for P-gp (**A-C**, purple), Bcrp (**D-F**, orange), and Mrp2 (**G-I**, green) in male (triangles) and female (circles) mice; each point represents the average across all images obtained from a single mouse. We found no statistically significant differences between males and females in (**A, D, G**) Pearson’s correlation coefficient (*r*), (**B, E, H**) Mander’s M_1_ coefficient (indicating the extent of occludin-labeled area overlapping with the transporter) or (**C, F, I**) Mander’s M_2_ coefficient (indicating the extent of transporter-labeled area overlapping with occludin). Data presented as mean ± SEM. T-tests, *p*<0.05 is significant.


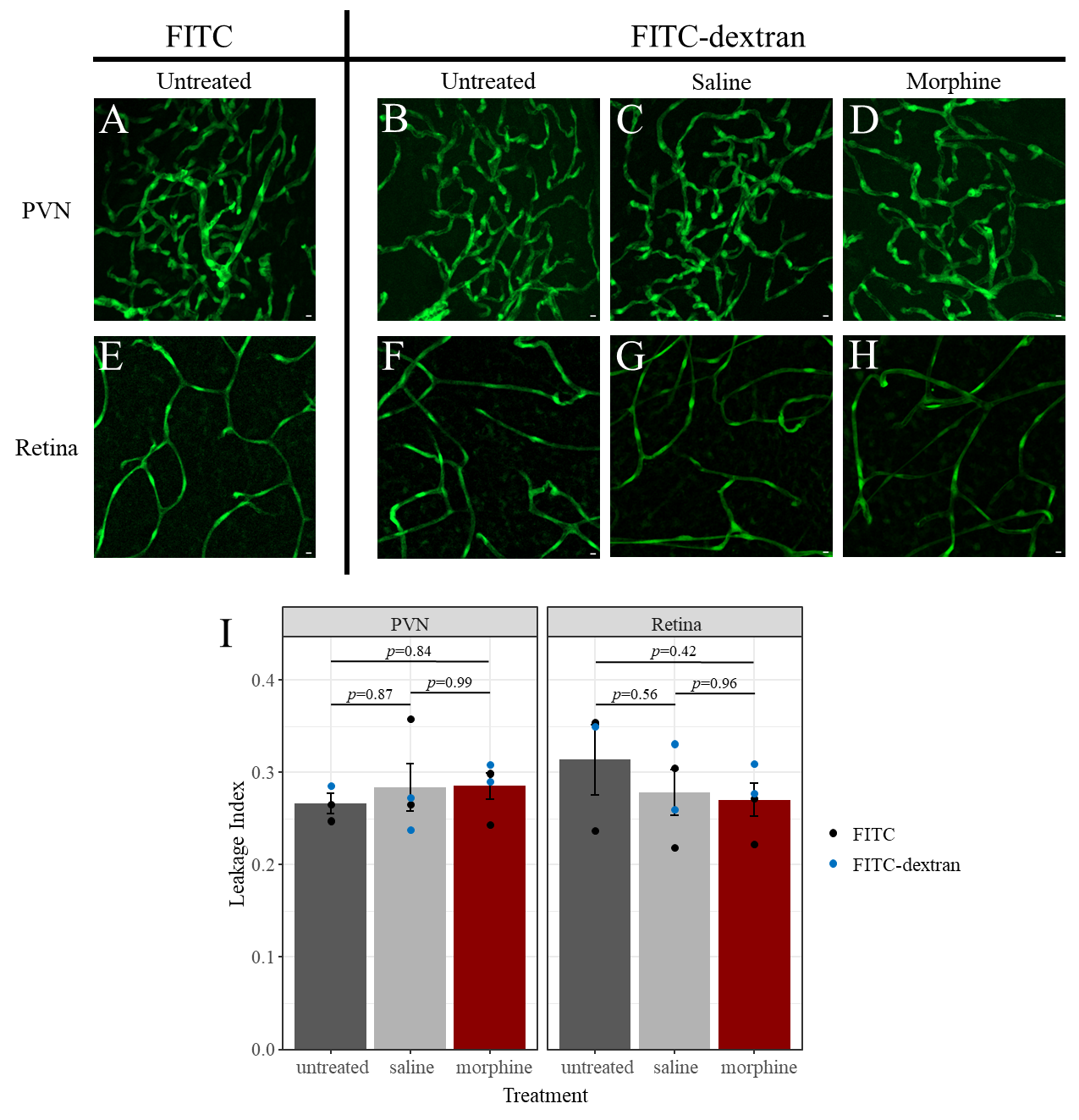


**Fig S3. FITC and FITC-dextran extravasation is similar at the BBB and BRB, independent of treatment.** Fluorescence imaging of FITC (**A**, **E**) and FITC-dextran extravasation (**B-D**, **F-H**) resulted in similar leakage indices in all assessed groups (**I**). Leakage index was calculated in the densely vascularized PVN (**A-D**) and in retinal capillaries (**E-H**), and is defined as the average intensity outside of vessels divided by the average intensity inside vessels. We found no differences in leakage index between mice that received chronic morphine treatment (20 mg/kg, twice daily, 6 d; n=4; **D**, **H**), vehicle-treated mice (saline, twice daily, 6 d; n=4; **C**, **G**) and untreated mice (n=3; **A**-**B**, **E**-**F**). Scale bars = 5 μm. Data are presented as the mean ± SEM. Two-way ANOVA with Tukey post-hoc adjustments found no significant differences (*p*<0.05).


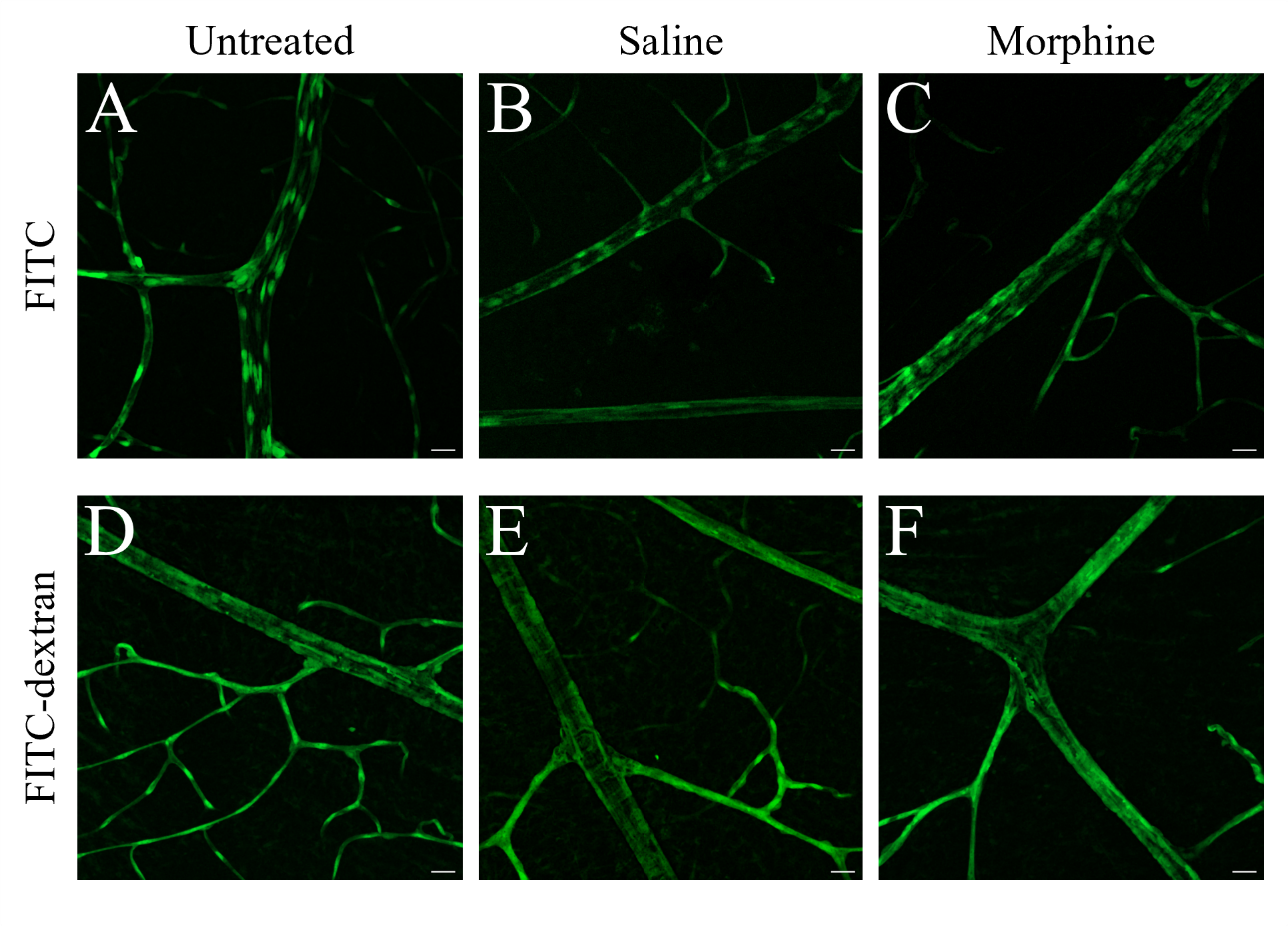


**Fig S4. Fluorescence extravasation in retinal arterioles is unchanged by morphine treatment.** Fluorescence imaging of FITC (**A**-**C**) and FITC-dextran extravasation (**D-F**) in first-order retinal arterioles in the superficial vascular layer in untreated (**A**, **D**), saline/vehicle-treated (**B**, **E**), and morphine-treated mice (**C**, **F**). Scale bars = 20 μm.
